# Effect of digital psychoeducation and peer support on the mental health of family carers supporting individuals with psychosis in England (COPe-support): a randomised clinical trial

**DOI:** 10.1016/S2589-7500(22)00031-0

**Published:** 2022-04-01

**Authors:** Jacqueline Sin, Claire Henderson, Jack Elkes, Victoria Cornelius, Luke A Woodham, Rachel Batchelor, Tao Chen, Ana Maria Corredor, David Coughlan, Ranjita Dhital, Sian Evans, Ban Haider, Julia Heathcote, Sarah Mansfield, Aileen O'Brien, Mona Qassim, Juliet Sserunkuma, Clive H Travis, Elen Williams, Steve Gillard

**Affiliations:** a School of Health Sciences, City, University of London, London, UK; b Health Service & Population Research Department, King's College London, Institute of Psychiatry, Psychology & Neuroscience, London, UK; c South London and Maudsley NHS Foundation Trust, Maudsley Hospital, London, UK; d Imperial Clinical Trials Unit, School of Public Health, Imperial College London, London, UK; e St George's, University of London, Centre for Technology in Education, London, UK; f Department of Public Health, Policy & Systems, Institute of Population Health, The University of Liverpool, Liverpool, UK; g South West London and St George's Mental Health NHS Trust, Springfield University Hospital, London, UK; h Arts and Sciences Department, University College London, London, UK; i Sussex Partnership NHS Foundation Trust and Oxford Cognitive Therapy Centre, Newhaven, UK; j Berkshire Healthcare NHS Foundation Trust, Bracknell, UK; k Specialist Services, Improving Access to Psychological Therapies, East London NHS Foundation Trust, London, UK; l Paranoidschizophrenia.co.uk, Bedford, UK; m Locum GP, London, UK

## Abstract

**Background:**

Psychoeducation delivered face-to-face is effective in alleviating mental health morbidities in family carers of individuals with psychosis. However, research in such interventions delivered online is scarce. We evaluated the effectiveness of a digital multicomponent intervention—COPe-support—in improving carers' mental wellbeing and caregiving-related outcomes.

**Methods:**

In this two-arm, individually randomised, superiority trial, people aged 18 years or older who provided at least weekly support in any format for a relative or close friend affected by psychosis across England were randomly assigned (1:1) to either COPe-support or a passive online information resource using an independent online system. Participants were recruited through 30 mental health UK National Health Service trusts. The study team were masked to allocation and assessment of outcomes as all data collection took place online. Participants had access to either condition for 40 weeks and were advised to spend at least half an hour per week over the initial 20 weeks to go through materials at their own pace and to allow time to integrate knowledge and skills learned into practice. It was not feasible to mask participants or the online facilitator to intervention allocation. COPe-support provided psychoeducation on psychosis-related caregiving strategies and forums with professionals and other carers, and the control intervention comprised a passive online information resource. The primary outcome at 20 weeks was mental wellbeing measured by the Warwick-Edinburgh Mental Wellbeing Scale (WEMWBS; minimally clinically important difference [MCID] 3). This trial is registered with ISRCTN, 89563420.

**Findings:**

Between March 1, 2018, and Feb 14, 2020, 407 participants were randomly assigned, with 204 allocated to COPe-support and 203 allocated to control. The participants (mean age 53·1 years, SD 13·2) were mostly female (330 [81%] of 407 participants) and White (359 [88%] of 407 participants). 346 (85%) of 407 participants provided primary endpoint data, 174 (85%) of 204 participants in the COPe-support group and 172 (85%) of 203 participants in the control group. The mean WEMWBS score at 20 weeks was 44·5 (SD 8·31) for the COPe-support group and 43·3 (9·19) for the control group. We found no evidence of a difference in wellbeing between the two groups (adjusted mean difference 0·37, 95% CI –1·14 to 1·88; p=0·63). In the COPe-support group, 106 (52%) of 204 participants met the complier definition of a minimum of two logins in separate weeks. The complier average causal effect analysis increased the difference in WEMWBS scores (adjusted difference 0·83, 95% CI –1·45 to 3·11; p=0·47), but this was lower than the MCID. There were no adverse events.

**Interpretation:**

Our findings did not support the use of COPe-support over a passive online information resource. However, further research to optimise digital interventions adjunctive to face-to-face support for carers remains important.

**Funding:**

National Institute for Health Research.

## Introduction

Worldwide, a large proportion of people provide substantial yet unpaid support and care to family members or friends living with a long-term illness. In the UK, about 1·5 million people are carers supporting an individual with mental ill health.^1^, ^2^, ^3^ Psychosis, including schizophrenia, is the most common severe mental illness.^1^, ^2^ As the onset of schizophrenia peaks in late teenage years or early adulthood, individuals with psychosis often require long-term treatment and support across a range of life domains.^1^, ^2^, ^3^, ^4^, ^5^ Carers play a crucial part in sustaining their loved ones' community-based treatment and promoting their recovery.^2^, ^3^, ^4^, ^5^ However, caregiving negatively impacts carers' health and wellbeing.^2^, ^3^, ^4^, ^5^

**Research in context**

**Evidence before this study**

We did two systematic reviews at the outset of the trial in 2016, one focusing on psychoeducation interventions for family carers of individuals with psychosis or schizophrenia delivered using any medium, and the second focusing on interventions delivered via the internet. For both reviews, we searched ten databases that cover medical and health publications (MEDLINE, PsycInfo, CINAHL, Embase, Web of Science, ASSIA, Cochrane Central Register of Controlled Trials, National Institute for Health Research—Health Technology Assessment database, Database of Abstracts of Reviews of Effect, and NHS Economic Evaluation Database) and grey literature (ProQuest dissertations and theses) for studies published in English and Chinese from database inception to Dec 1, 2016. The search terms comprised subject headings, synonyms, and free-text search terms to identify (digital/e- or m-/web or internet-) psychoeducation interventions for family/informal/unpaid carer* for people affected by schizophreni* or psycho*. Searches were limited to randomised controlled trials. Through both reviews, we identified 32 randomised trials, including 2858 family carers; only one trial that reported an entirely digital intervention with 21 carers (and 31 patients) was identified. We did a meta-analysis, considering a range of mental health and caregiving-related outcomes. With relevance to this study, pooled results showed that psychoeducation, using a textual or face-to-face delivery format, was superior in reducing carers' global morbidities (standardised mean difference [SMD] –0·230), perceived burden (SMD –0·434), negative caregiving experiences (SMD –0·210), and expressed emotion (SMD –0·161), compared with usual care. Effects on carers' mental wellbeing were non-significant but data for this outcome were retrieved from just two trials and 184 participants. Only one trial investigated carers' quality of life as an outcome. The search that focused on trials of digital interventions targeting family carers was updated on Dec 20, 2020, identifying an additional trial investigating a digital self-management intervention that was facilitated by carer peer workers, but carers' mental wellbeing was not measured as an outcome. The search was updated on Aug 2, 2021, and the evidence base remains valid.

**Added value of this study**

To our knowledge, this is the first fully randomised trial to report the clinical efficacy of a digital psychoeducation intervention that provides quality health information and network support from professionals and carers as peers. This trial provides clear evidence that a multi-component psychoeducation intervention delivered entirely online is not effective in improving carers' mental wellbeing compared with a passive online information resource, at 20 weeks. There was a significant increase in mental health knowledge at 40 weeks in the intervention group. The trial was indicative of a small-to-moderate increase in wellbeing, quality of life, and positive caregiving experience, and a small reduction in negative caregiving experience and expressed emotion, at both 20 weeks and 40 weeks, but these changes were statistically non-significant. Effects on mental wellbeing at 20 weeks were greater in participants with higher use, although this finding was not significant. Study participant retention was high at 20 weeks and 40 weeks, with no adverse events or side-effects reported, indicating it is feasible to deliver psychoeducation safely via the internet.

**Implications of all the available evidence**

Our study results suggest that a digital multicomponent psychoeducation intervention has no significant effect on carers' mental wellbeing and quality of life, outcomes that have long been under-researched in the field of psychoeducation intervention effectiveness. In terms of caregiving-related outcomes that are more commonly reported in existing trials, such as caregiving experience and expressed emotion, our digital intervention was no more effective than was the control intervention. Our trial did not measure morbidities such as depression, anxiety or distress, or perceived burden, for which a positive effect has been identified in systematic reviews on conventional psychoeducation interventions delivered to family carers. Participant recruitment, retention, and completion in the trial were satisfactory. Notwithstanding the need for good evidence of clinical effectiveness and cost-effectiveness, the implication for policy is that digital psychoeducation intervention alone should not be commissioned for carers supporting an individual with psychosis with an expectation of a significant effect on mental wellbeing and other health outcomes. However, we note that participants' usage of the digital intervention—in particular, posting on interactive forums—was highly heterogeneous and, overall, far below the recommended weekly login for half an hour. Considering the projected increase in the size of the carer population, it is not possible to meet these needs in primary care or specialist mental health services without using the potential digital technologies offer. Further research to optimise the potential of digital technologies in delivering effective support, possibly adjunctive to professional-led and face-to-face services, including strategies to optimise engagement and increase usage, remains important.

Clinical guidelines recommend psychoeducation to provide information on psychosis and its management for carers.^1^, ^6^, ^7^ Previous systematic reviews of clinical trials showed that psychoeducation that targeted carers as primary participants, with or without the patients' presence or involvement in the intervention, reduced carers' mental illness morbidities and caregiving burden.^1^, ^2^, ^4^, ^5^ These changes mediate carers' caregiving capacity,^3^, ^4^, ^5^, ^7^, ^8^ translating to reduced relapse and better prognosis for patients.^4^, ^5^, ^7^ However, implementation of such strategies is restricted^2^, ^4^, ^9^ because of insufficient resources in routine health services, and carers' difficulties in attending sessions among multiple commitments.^2^, ^9^ Although the internet offers a novel, accessible, and self-paced approach to deliver mental health interventions that addresses some of these barriers,^10^, ^11^, ^12^ only one pilot study^13^ and one definitive^14^ randomised trial have evaluated such interventions in the field of psychosis caregiving to date.

Effective psychoeducation interventions for carers often comprise evidence-based health information and strategies to address commonly encountered challenges in psychosis caregiving, led by professionals and using a group format to enable exchanges of experiences and support among peers.^4^, ^5^, ^11^ Although most psychoeducation interventions tend to last for around 20 weeks, given their goals in imparting cognitive and behaviour changes in carers, previous meta-regression revealed no significant associations between intervention duration or contact time and outcomes.^4^ We previously developed and tested the usability of an entirely web-based multicomponent intervention called COPe-support.^15^, ^16^ This study examined whether COPe-support improved carers' mental wellbeing and health-related and caregiving-related outcomes, compared with a passive online information resource as control.

## Methods

### Study design and participants

In this randomised trial, we used a two-arm, individually randomised, superiority design that compared COPe-support with a passive online information resource. We did an internal pilot trial to test the trial procedures, recruitment, and retention, that ran for the first 12 months of the 30-month trial duration. The pilot stop–go criteria were: to recruit at least a third of the sample size and to retain at least 80% of participants, with over 80% of them having activated their logins. A detailed trial protocol has been previously published.^17^ This study has been reviewed and approved by South Central—Oxford C Research Ethics Committee (reference 18/SC/0104) and the UK National Health Service (NHS) Health Research Authority (reference IRAS 240005). Substantial patient, carer, and public involvement activities underpinned the design and conduct of the study. We have eight patient, carer, and key stakeholder members among our 14-membered Project Reference Group, which oversaw the study design, identified the outcomes and measures used in the study, and supported the study delivery, data analysis, and dissemination of the study results.^15^

The trial included relatives and close friends aged 18 years or older who provided at least weekly support in any format for the cared-for person who had psychosis,^1^ had daily access to the internet including emails, and were able to communicate in English. Both the carers and the cared-for person were required to reside in England during the study period. To avoid a clustering effect, potential participants who had another relative or friend who shared the caring role for the same cared-for person already enrolled in the study were excluded.

Participants were recruited through 30 mental health NHS trusts (provider organisations) across England. We advertised through flyers and posters, asking health-care workers to inform potentially eligible carers, national and local voluntary organisations that provide support for carers, our study website, and social media communications with relevant organisations.

Potentially suitable participants completed an online eligibility screening process through our research website. Queries were resolved and further information provided through phone or online discussion with the study coordinator. Those eligible provided online written informed consent.

### Randomisation and masking

We used an independent online system to randomly assign participants (1:1), stratified by gender (male *vs* female) and recruitment cohort (cohort 1 to 6), to either COPe-support or a passive online information resource. We used a permuted block randomisation scheme that included randomly selected sized blocks of two and four. Over the study duration, we scheduled participants into six cohorts, placed 4 months apart to ensure optimal numbers (ie, ≥20 but ≤60 of participants in each arm).^4^, ^11^ Randomisation was done in order of baseline data collection in the 4 months recruitment time prior to each cohort starting. The study team were masked to allocation and assessment of outcomes as all data collection took place online. It was not feasible to mask participants or the online facilitator to intervention allocation. Statistical analyses were done masked to allocation.

### Procedures

Participants received an email that provided unique login details to access the allocated intervention, together with instructions to download the free app (Apple or Android), which enabled use through smart phones or tablets, in addition to computers via web browsers. Participants had access to either condition for 40 weeks and were advised to spend at least half an hour per week over the initial 20 weeks to go through materials at their own pace and to allow time to integrate knowledge and skills learned into practice. An experienced mental health nurse (JSi) acted as the online facilitator who monitored and moderated all the interactive functions. The online facilitator sent weekly updates via the intervention announcement function, which generated an email automatically to participants to promote engagement. For security and anonymity, participants were required to follow ground rules, including the use of a self-chosen pseudonym and to not give any of their or their cared-for person's identifiable details on the platform.^15^, ^16^

COPe-support was codesigned and coproduced with people with lived experiences of psychosis or caring experience, and key stakeholders working in the field, through a participatory research study.^15^ Our considerations of the intervention content and key ingredients, duration and intensity (ie, 20 weeks), and facilitation strategies (eg, moderation and anonymous participation) had also been informed by our theoretical development work.^4^, ^11^ During the intervention-build process, 24 carers independent from the study reviewed and gave feedback on the early versions of the prototype.^15^ A mixed methods usability study incorporating a think-aloud test and remote usability test was done with 20 carers over 3 months, from Nov 1, 2017, to Jan 31, 2018, to establish the feasibility of delivering COPe-support via the internet (appendix pp 13–14).^16^

COPe-support was a multimedia, interactive intervention that provided psychoeducation and network support with peers and professionals, delivered through the web-based learning environment Canvas. In addition to a home page, which provided a menu, navigation video, and guide, the intervention comprised 12 sections. These included psychoeducation on psychosis, its treatment, and related caring issues; wellbeing promotion information and exercises; an ask the experts online forum, where participants could ask for advice from 14 experts, including multidisciplinary clinicians, welfare benefits advisors, and people using mental health services; a peer-to-peer forum, where participants could exchange views and support; a resources for carers section that provided extensive links to relevant external resources (eg, statutory and professional bodies, charities, books, and online information sites); and a support weblink, for participants to seek technical or emotional support if necessary. There was no prescribed order or sequence on the content; instead, participants were encouraged to pick the content specific to their own needs and caring situations. Participants were able to initiate posts and respond to both ask the experts and peer-to-peer forums or visit both forums passively (ie, reading posts).

A web-based information resource run on a parallel Canvas platform acted as the control, which had an identical presentation and format to the COPe-support platform. The control platform comprised a home page, support function, and all the information provided under resources for carers in the COPe-support platform. There were no interactive elements included in the passive online information resource platform, similar to other information websites as the most common provision within wider usual care. To optimise retention, control group participants were given access to COPe-support for 20 weeks, after completing 40-week follow-up data collection.

All participants had unrestricted access to usual care, which commonly comprised information and advice sought from primary care or NHS mental health service or voluntary organisations. As usual care for carers varies geographically and based on participants' circumstances, we collected health, social, voluntary, and other service use data from participants.

To assess mental wellbeing, we used the Warwick-Edinburgh Mental Wellbeing Scale (WEMWBS).^18^ The WEMWBS has been widely used to measure positive mental health at the population level;^19^ its scores range from 14–70 with higher scores indicating better wellbeing,^18^ and a change of 3 points represents the minimally clinically important difference (MCID).^19^ Quality of life was measured using the EQ-5D-5L visual analogue scale (VAS), on which higher scores represent better subjective judgement of health state.^20^ Mental health knowledge was measured using the Mental Health Knowledge Schedule (MAKS); higher scores indicate better stigma-related mental health knowledge.^21^ Appraisal of caregiving experience was measured using the Experience of Caregiving Inventory (ECI), which has positive subscales for which higher scores indicate better experience, and negative subscales with higher scores indicating poorer experience.^22^ Carers' wellbeing and satisfaction with support was measured using the Carer Wellbeing and Support Scale (CWS); higher wellbeing subscale scores indicate higher wellbeing, and higher support subscale scores with reversed scoring indicate worse satisfaction.^23^ Carers' expressed emotion in terms of criticism and over-involvement was measured with the Family Questionnaire (FQ), on which higher scores indicate higher expressed emotion.^24^

Study outcomes were assessed at baseline, mid-treatment (10 weeks), 20-week follow-up (primary endpoint), and 40-week follow-up. All primary and secondary outcomes were self-report measures completed by participants online. At baseline, participants were asked to provide demographic data and information on their caregiving situation, such as time spent caregiving per week, relationship with the cared-for person, and minimal, non-identifiable information about the cared-for person, comprising age, gender, specific psychotic disorder type, and time since illness onset.^17^

We prespecified use data at 20 weeks to be automatically recorded by the online platform,^17^ including number of logins in separate weeks, weekly and total page views over 20 weeks, total time spent on the platform in min, and mean time spent per page view, for both groups. For the COPe-support group, the numbers of posts per participant made to each forum were recorded. These measures are the most recorded digital health intervention use metrics reported in similar studies.^10^, ^11^, ^12^, ^13^, ^14^

The perceived acceptability of COPe-support was obtained through individual interviews after 40 weeks of follow-up with 20% of the intervention participants. These data have been reported separately.^25^

### Outcomes

The primary outcome was mental wellbeing at 20 weeks, assessed by the WEMWBS.^18^

Secondary outcomes at 20-weeks were: quality of life, mental health knowledge, appraisal of caregiving experience, carer wellbeing and satisfaction with support, and carers' expressed emotion in terms of criticism and over-involvement.

For safety monitoring, in addition to adverse events and serious adverse events, we devised a category labelled unintended consequences, to denote incidents of access interruptions to the interventions. All safety events were documented, categorised, and evaluated by an independent Trial Steering Committee (appendix pp 23–24).^17^

### Statistical analysis

To detect an MCID of 3 points for the between-group difference in WEMWBS score,^20^ assuming a SD of 9,^26^ a sample size of 360 participants was required with α=0·05 (two-tailed) at 80% power, accounting for 20% dropout.

Our primary estimand was the effect of COPe-support for all participants regardless of use (treatment policy estimand),^27^ including participants with at least one post-baseline assessment in their allocated groups (intention-to-treat principle). We used a linear mixed model to estimate the mean difference in WEMWBS score between arms at 20 weeks. The model included baseline score, three indicators for each timepoint (10 weeks, 20 weeks, and 40 weeks), an indicator for intervention allocated, a timepoint and intervention interaction, gender (stratification variable), parent (yes *vs* no), and living with the cared-for individual (yes *vs* no) as fixed effects, with cohort and participant as random effects. The model was fitted using restricted maximum likelihood and assumptions were checked through residual plots. A further analysis estimated the intervention effect for the subsample of intervention compliers (intervention efficacy estimand). We predefined compliers as participants in the COPe-support group who made at least two logins in separate weeks by week 20 (ie, those who had chosen to return to the intervention after their initial successful login activation). We performed a complier average causal effect (CACE) analysis^28^ using a two-stage least squares regression with randomisation as the instrumental variable. We also did a post-hoc CACE analysis to assess intervention effects of different use thresholds as alternative complier definitions.

We did a sensitivity analysis to explore the effect of missing data and departures from the missing-at-random (MAR) assumption on the primary estimand, using controlled multiple imputation. Imputations were done separately within each intervention arm using variables in the primary model and auxiliary variables identified as strongly associated in previous work.^3^ A further sensitivity analysis was also done using multiple imputation across every timepoint to include all participants.

The intervention difference for all secondary outcome measures was estimated as the treatment policy estimand, using linear regression models including intervention arm, baseline score, gender, and cohort. The analysis was done separately at each timepoint (10 weeks, 20 weeks, and 40 weeks), with the primary focus at 20 weeks. Incidents of adverse events, serious adverse events, and unexpected consequences were tabulated showing number of events and number of participants per arm.

Analyses were done with Stata version 15. The trial was registered prospectively with ISRCTN, ISRCTN89563420.

### Role of the funding source

The funder of the study had no role in study design, data collection, data analysis, data interpretation, or writing of the report.

## Results

**Figure 1:**
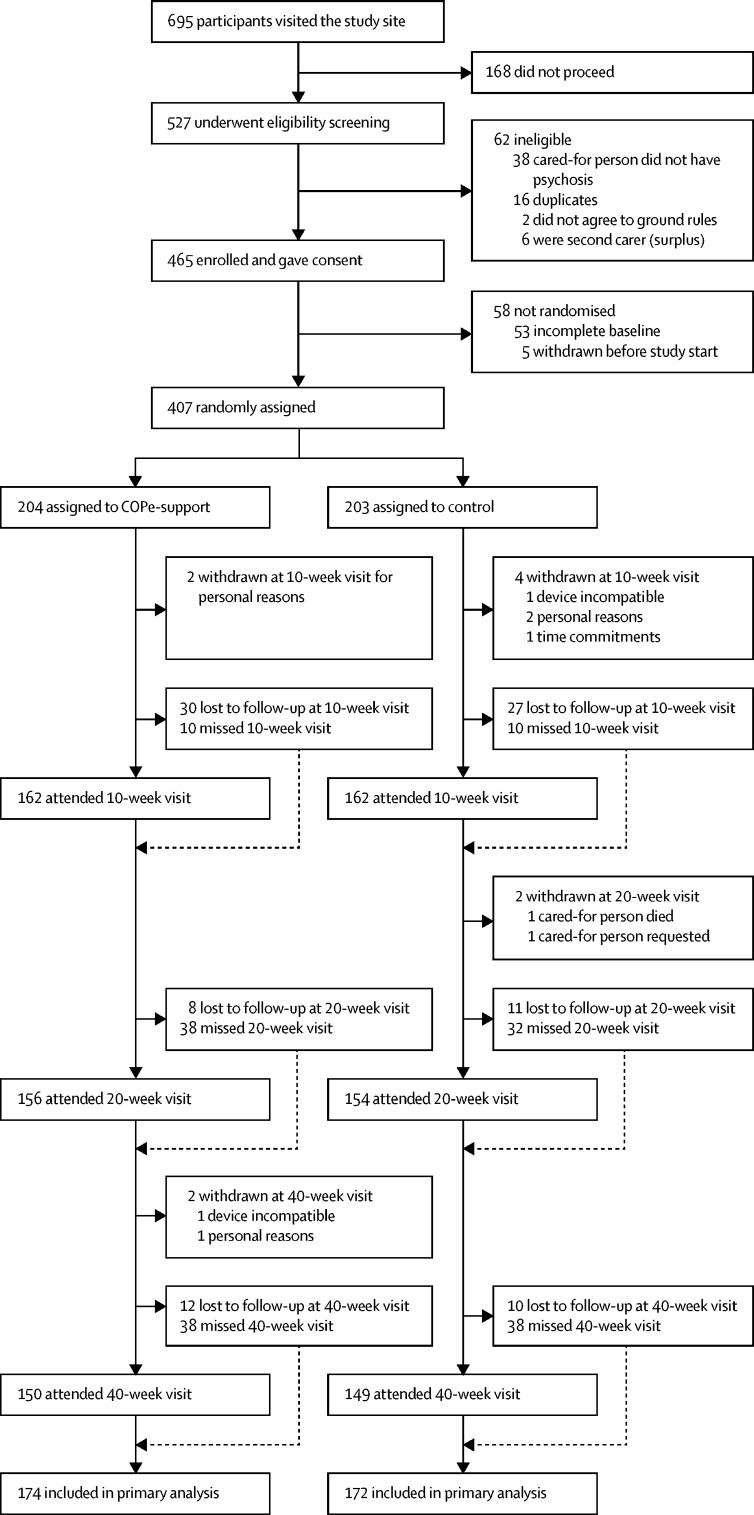
Trial profile A participant who missed a visit did not provide any data at the given timepoint; a participant who attended a visit was not required to have completed all outcome data. The number of participants included in the primary analysis were those with at least one post-baseline assessment in their allocated groups.

Between March 1, 2018, and Feb 14, 2020 (the study recruitment period), there were 695 visits to the online study enrolment platform. 527 people undertook eligibility screening, with 465 confirmed as eligible for the study (figure 1). 189 of the participants were recruited into the internal pilot. Criteria for the internal pilot were met and the trial steering committee approved the trial to progress. 407 participants completed baseline assessment and were randomly assigned, with 204 allocated to COPe-support and 203 allocated to control. The last participant completed their final visit on Oct 31, 2020. Four (2%) of 204 participants withdrew from the intervention group and six (3%) of 203 participants withdrew from the control group.

Baseline characteristics were similar between the two study groups (table 1). The mean participant age was 53·1 years (SD 13·2), 330 (81%) of 407 participants were women, 258 (63%) were parents, 359 (88%) were White, and 197 (49%) had been caring for less than 5 years. Participants were recruited from all regions of NHS mental health services in England (North East, North West, South East, South West of England, Yorkshire, Midlands, and East England). During the first 20 weeks of the study, 355 (87%) of 407 participants activated their logins—175 (86%) of 204 participants in the intervention group and 180 (89%) of 203 participants in the control group. The COPe-support group logged in a median of 4·0 times (IQR 2·0–7·0), with a median usage time of 69·7 min (17·3–176·1). Control participants logged in a median of 2·0 times (1·0–4·0), with a median usage time of 20·4 min (9·1–38·8; appendix p 63).

**Table 1:** Demographic and caregiving characteristics of study participants at baseline

		**COPe-support (n=204)**	**Control (n=203)**
Age, years		53·7 (13·3)	52·5 (13·1)
Gender			
	Male	39 (19%)	38 (19%)
	Female	165 (81%)	165 (81%)
Race			
	White	178 (87%)	181 (89%)
	Mixed	7 (4%)	8 (4%)
	Asian	10 (5%)	6 (3%)
	Black	8 (4%)	6 (3%)
	Other	1 (<1%)	2 (1%)
Employment status			
	Full time	68 (33%)	63 (31%)
	Part time	40 (20%)	43 (21%)
	Full or part-time education	6 (3%)	4 (2%)
	Unemployed	5 (2%)	3 (1%)
	Permanently disabled or sick	6 (3%)	11 (5%)
	Retired	50 (25%)	45 (22%)
	Looking after family or home	25 (12%)	27 (13%)
	Other	4 (2%)	7 (3%)
Highest education level achieved			
	Secondary	50 (25%)	49 (24%)
	A level	11 (5%)	17 (8%)
	University	67 (33%)	65 (32%)
	Post-graduate	54 (26%)	33 (16%)
	Apprenticeship	16 (8%)	24 (12%)
	Professional qualification	6 (3%)	15 (7%)
Marital status			
	Single	40 (20%)	41 (20%)
	Married or cohabiting	140 (69%)	138 (68%)
	Divorced	23 (11%)	22 (11%)
	Other	1 (<1%)	2 (1%)
Relationship with cared-for person			
	Parent	129 (63%)	129 (64%)
	Spouse or partner	46 (23%)	41 (20%)
	Child	9 (4%)	12 (6%)
	Sibling	11 (5%)	12 (6%)
	Other relative	0	1 (<1%)
	Friend	9 (4%)	8 (4%)
Living arrangement			
	With cared-for person	111 (54%)	116 (57%)
	Not with cared-for person	93 (46%)	87 (43%)
Duration of care, h per week			
	1–9	60 (29%)	50 (25%)
	10–19	44 (22%)	33 (16%)
	20–34	23 (11%)	34 (17%)
	35–49	15 (7%)	26 (13%)
	≥50	62 (30%)	60 (30%)
Age of cared-for person, years		35·5 (14·2)	34·5 (13·7)
Sex of cared-for person			
	Female	72 (35%)	80 (39%)
	Male	132 (65%)	123 (61%)
Time since cared-for person's illness onset, years			
	0–5	102 (50%)	95 (47%)
	≥5–10	26 (13%)	38 (19%)
	>10	76 (37%)	70 (34%)

Data are mean (SD) or n (%).

**Figure 2:**
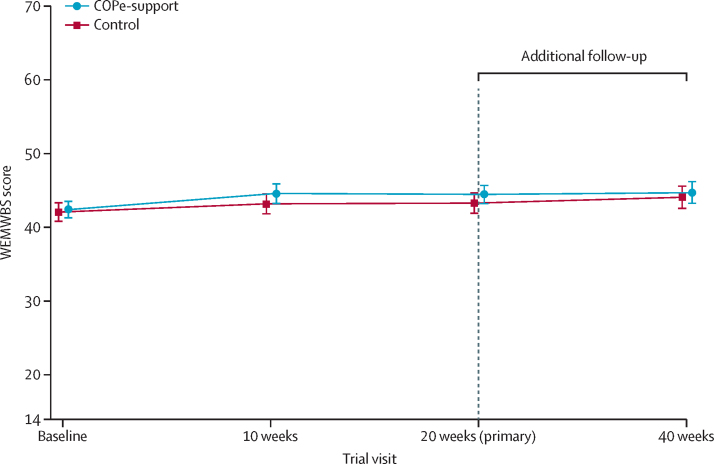
Unadjusted mean WEMWBS score across the four timepoints by treatment arm Error bars indicate 95% CIs. The vertical dotted line indicates the start of the additional follow-up period. WEMWBS=Warwick-Edinburgh Mental Wellbeing Scale.

The primary analysis included 174 (85%) of 204 participants in the COPe-support group and 172 (85%) of 203 participants in the control group (participants with at least one post-baseline assessment in their allocated groups). The mean WEMWBS score at 20 weeks was 44·5 (SD 8·31) in the COPe-support group and 43·3 (9·19) in the control group (figure 2). The adjusted between-arm mean difference in WEMWBS score was 0·37 (95% CI –1·14 to 1·88; p=0·63; table 2). A sensitivity analysis to explore the effect of missing data resulted in an estimated between-group range in WEMWBS score from 0·09 to 0·75 at 20 weeks (appendix p 64). A further supplementary analysis including all participants randomly assigned, showed a between-arm difference in WEBWMS score of 0·24 (95% CI –1·43 to 1·91) at 20 weeks*.* This additional analysis was consistent with our original conclusions.

**Table 2:** Analysis of primary and secondary efficacy outcomes at 20 weeks

	**COPe-support**		**Attention control**		**Adjusted treatment difference (95% CI)**	**p value**
	n	Mean (SD)	n	Mean (SD)		
**Primary outcome**						
Warwick-Edinburgh Mental Wellbeing Scale	156	44·5 (8·31)	151	43·3 (9·19)	0·37 (−1·14 to 1·88)	0·63
**Secondary outcomes**						
Mental Health Knowledge Schedule	156	24·1 (2·56)	151	23·7 (2·86)	0·39 (−0·14 to 0·91)	..
ECI negative	155	96·6 (35·50)	148	100·5 (34·61)	−3·39 (−8·26 to 1·49)	..
ECI positive	155	30·2 (7·76)	148	29·8 (7·68)	0·38 (−0·96 to 1·72)	..
EQ-5D 5-level visual analogue scale	154	70·2 (17·08)	147	69·0 (21·22)	0·52 (−2·79 to 3·83)	..
Family Questionnaire	154	48·6 (10·66)	147	49·0 (10·78)	−0·20 (−1·80 to 1·39)	..
CWS-support	154	20·1 (11·33)	148	21·2 (11·39)	−1·04 (−3·09 to 1·02)	..
CWS-wellbeing	154	80·7 (28·24)	148	78·8 (28·36)	−0·91 (−5·29 to 3·46)	..

ECI=Experience of Caregiving Inventory. CWS=Carer Wellbeing and Support Scale

The adjusted mean difference between the study groups at 10 weeks was 1·05 (95% CI –0·43 to 2·53; p=0·17). At 40-week follow-up, the adjusted between-arm mean difference was 0·37 (95% CI –1·16 to 1·89; p=0·64; table 3).

**Table 3:** Analysis for primary and secondary efficacy outcomes at 10 weeks and 40 weeks follow-up

		**COPe-support**		**Attention control**		**Adjusted treatment difference (95% CI)**	**p value**
		n	Mean (SD)	n	Mean (SD)		
**Primary outcome**							
Warwick-Edinburgh Mental Wellbeing Scale							
	Week 10	161	44·6 (8·77)	161	43·2 (9·34)	1·05 (−0·43 to 2·53)	0·17
	Week 40	149	44·7 (9·41)	149	44·1 (9·86)	0·37 (−1·16 to 1·89)	0·64
**Secondary outcomes**							
Mental Health Knowledge Schedule							
	Week 10	161	23·8 (2·90)	161	23·8 (2·92)	0·00 (−0·54 to 0·54)	..
	Week 40	149	24·0 (2·93)	148	23·4 (3·00)	0·64 (0·11 to 1·18)	..
ECI negative							
	Week 10	160	103·0 (34·56)	159	102·2 (32·27)	−0·46 (−4·64 to 3·71)	..
	Week 40	149	99·3 (37·62)	147	98·0 (36·10)	0·71 (−4·73 to 6·14)	..
ECI positive							
	Week 10	160	30·5 (8·21)	159	29·7 (7·16)	0·49 (−0·78 to 1·76)	..
	Week 40	149	29·8 (7·57)	147	29·8 (7·74)	−0·20 (−1·56 to 1·16)	..
EQ-5D 5-level visual analogue scale							
	Week 10	160	70·7 (17·16)	157	68·9 (19·79)	1·03 (−1·71 to 3·77)	..
	Week 40	149	68·6 (18·76)	146	69·7 (18·01)	−0·93 (−4·21 to 2·35)	..
Family Questionnaire							
	Week 10	160	49·5 (10·26)	157	49·3 (10·12)	−0·04 (−1·45 to 1·38)	..
	Week 40	149	49·0 (10·12)	147	48·3 (11·16)	0·42 (−1·29 to 2·13)	..
CWS-support							
	Week 10	160	20·4 (11·29)	157	20·7 (11·24)	−0·15 (−2·00 to 1·69)	..
	Week 40	149	20·5 (12·07)	147	20·4 (11·66)	−0·18 (−2·35 to 1·98)	..
CWS-wellbeing							
	Week 10	160	78·3 (28·17)	158	76·8 (26·98)	−0·93 (−4·85 to 2·98)	..
	Week 40	149	78·7 (28·52)	147	79·0 (29·32)	−2·13 (−6·84 to 2·59)	..

ECI=Experience of Caregiving Inventory. CWS=Carer Wellbeing and Support Scale.

The primary CACE analysis included 156 (76%) of 204 participants in the intervention group and 151 (74%) of 203 participants in the control group (participants who provided WEMWBS score data at 20 weeks). In the intervention group, 106 (52%) of 204 participants met the prespecified complier definition (ie, ≥2 weekly logins). The mean WEMWBS score for these participants at 20 weeks was 44·9, compared with 42·9 for the would-be compliers in the control group. The adjusted between-arm difference in WEMWBS score was 0·83 (95% CI –1·45 to 3·11; p=0·47), showing no evidence of a statistical difference between the study groups. The efficacy estimate was greater than the treatment policy estimand in favour of COPe-support, but not close to the MCID of 3. Post-hoc analyses using different complier definitions resulted in an estimated range of between-arm difference from 0·87 to 1·45, and the difference was greatest in those who had spent over 120 min on the COPe-support platform by 20 weeks (table 4). This additional analysis was consistent with our original conclusions.

**Table 4:** Complier average causal effect analysis for Warwick-Edinburgh Mental Wellbeing Scale score for different complier definitions

	Number of participants*	**Adjusted treatment estimate (95% CI)**
Weekly logins ≥2 (original)	96	0·83 (−1·45 to 3·11)
Weekly logins ≥2 and post ≥1	57	1·38 (−2·40 to 5·16)
Total activity >60 min	83	0·96 (−1·66 to 3·59)
Total activity >120 min	55	1·45 (−2·52 to 5·42)
Page views >100	92	0·87 (−1·50 to 3·24)

* Number of COPe-support participants who met the complier definition.

Results from the analysis of our secondary outcomes were similar to the results for the primary outcome (table 2). The adjusted 20-week between-arm difference was small for all secondary outcomes and there was no evidence of a significant difference. The point estimates for six of the measures we investigated were in favour of the COPe-support arm (MAKS, ECI positive and negative subscales, EQ-5D-5L VAS, FQ, and CWS-support) and one measure (CWS-wellbeing) was in favour of the control. At 40-week follow-up, similar results for all secondary outcomes, apart from mental health knowledge, were found; the intervention group had significantly higher MAKS (0·64; 95% CI 0·11–1·18; p=0·018) compared with the control group (table 3).

No adverse events or serious adverse events were reported. There were 21 unintended consequences reported by 20 participants: ten in the intervention group and 11 (by ten participants) in the control group. All unintended consequences were related to problems in activating logins or accessing the interventions.

## Discussion

In this study, among carers supporting an individual with psychosis, using COPe-support to provide web-based interactive psychoeducation and network support for 20 weeks did not significantly improve mental wellbeing compared with a passive online information resource. Similar results were found for all secondary outcomes at 20 weeks and 40 weeks, with the exception of stigma-related mental health knowledge, which reflected a more positive outlook on treatment effectiveness and on recovery. Although mental wellbeing and a range of caregiving-related and mental health outcomes improved among COPe-support participants, COPe-support was not superior to the control intervention.

Our trial results echoed those of a study of an online self-management intervention facilitated by trained peer workers for carers of people with psychosis in England.^14^ We propose several potential explanations for the results. First, overall use of COPe-support was below our recommended weekly 30 mins of use for 20 weeks; we did anticipate participants' use would be heterogeneous and less than regular, depending on needs. Heterogeneous use and adherence has been frequently observed in digital mental health interventions, in particular in self-guided ones.^10^, ^11^, ^12^, ^13^, ^14^, ^29^ Although face-to-face psychosocial interventions commonly have in-built engagement sessions that have been shown to mediate therapeutic alliance, adherence, and effects,^4^, ^5^, ^30^ online interventions such as COPe-support might fall short in engagement with participants, relying solely on online communciations, and therefore hampering ongoing content delivery.^10^, ^11^, ^12^, ^29^ Our CACE analysis showed a greater effect in participants with higher use, but this difference was not significant. This finding raises the possibility that the absence of a significant effect could have been partly because of low or no minimally sufficient treatment dosage. However, there is insufficient research to indicate the required compliance threshold.^11^, ^29^ Second, although the effectiveness of face-to-face psychoeducational interventions in reducing psychological distress and burden is well established,^4^, ^5^ the pathways to positive effects on carer mental health remain unclear. Third, although participants reported feeling better supported through COPe-support, it is possible that they did not observe clinical improvement in the people they were caring for; therefore, their own outcomes did not change. Almost all the participants were recruited through mental health services, indicating that the carers were supporting an individual receiving care at the time of the study. This fact might imply that participants' need for support was high and beyond what COPe-support was designed for. Fourth, participants across groups had access to health information resources and usual care, potentially contributing to a larger control group effect. Previous conventional psychoeducational intervention trials commonly used waitlists or usual care as a comparator and so a tougher test of effect was applied in this study.^4^, ^5^

The strengths of this study were its large sample size, broad inclusion criteria that considered the varied nature of caregiving in psychosis (such as different relationships between carers and cared-for people and time spent on caregiving), a web-based active control group to reduce confounding factors inherent in technology or delivery medium, and use of methods designed to reduce the risk of bias, such as participant-reported outcome measures, statistical analysis done masked to allocation, intention-to-treat analyses, and a pre-registered statistical analysis plan. Our study did have some limitations. Our results might be more generalisable to individuals who are similar to the study sample, most of whom were women, White, and used internet communications regularly. We recruited carers as participants across England where, although health services share the same systems and clinical guidelines,^1^ variation in care and resources available for carers across areas does exist. Although the random assignment should have removed any allocation bias, we did not prespecify the areas in which carers or their cared-for people resided as a stratification factor. Similar to many other psychotherapy trials, participant masking was not possible, which might have led to bias by expectation, considering that all outcomes were self-reported.

Psychoeducation, delivered as a group programme that facilitates exchanges of experiential knowledge and support between peers, is among the most effective interventions recommended for carers supporting an individual with psychosis.^1^, ^2^, ^4^, ^5^, ^7^ Paradoxically, implementation of such interventions is limited. Self-guided digital interventions, such as COPe-support, offer full user autonomy and require minimal input from health-care professionals. A high proportion of carers remained engaged with the intervention for 40 weeks, with no adverse events reported over the 30-month study duration. These findings suggest that digital technology can be used effectively to meet carers' needs for psychoeducation and network support across a vast geographical area. Our study findings on improved stigma-related mental health knowledge among carers suggest that COPe-support might have had benefits in relation to knowledge rather than in relation to wellbeing and health; we will explore this hypothesis further in the qualitative analysis of participant interviews. Further research is also needed to explore the effects of peer workers or peer researchers instead of clinicians acting as online forum facilitators or study coordinators, in both usage of and nature of interactions. COPe-support, adapted with better digital engagement strategies that promote higher use and as an adjunct to some face-to-face support from local services, might have the potential to fill an important gap in delivering high-quality psychoeducation to carers. Considering the strong association between carers' own wellbeing and their caring capacity, which inevitably affect the recovery of the person they care for,^3^, ^8^, ^9^ interventions targeting carers, as both users and providers of care, can have far-reaching effects. Furthermore, with the implementation of digital interventions, accelerated in light of the COVID-19 pandemic to overcome face-to-face contact restrictions, further research is warranted to investigate strategies to optimise use and effects of carer-focused digital interventions. With the projected increase in the carer population, many of whom are unable to attend or have no access to in-person interventions, it is not possible to meet these needs in primary care or specialist mental health services without exploiting the potential of digital technologies.

In conclusion, our results show that for carers supporting an individual with psychosis, COPe-support providing online psychoeducation and support from professionals and peers was not superior to a high-quality passive information resource in improving mental wellbeing at 20 weeks. Our findings do not support the use of COPe-support in its current format rather than a passive online information resource. Considering the projected increase in the carer population and demand for support, delivering evidence-based interventions via the internet adjunctive to face-to-face services remains a potentially viable option.

## Data sharing

Individual participant data that underlie the results reported in this Article, after de-identification (text, tables, figures, and appendices), will be shared by the corresponding author upon reasonable request for academic and research purposes, and subject to Data Sharing Agreements. The statistical analysis plan, including the analytical code for the primary analysis, is available in the appendix (pp 54–55).

## Declaration of interests

We declare no competing interests.
