# Supplementary material for: Effect of digital psychoeducation and peer support on the mental health of family carers supporting individuals with psychosis in England (COPe-support): a randomised clinical trial
Source: Lancet Digit Health. 2022 Apr 1;4(5):e320–9. doi: 10.1016/S2589-7500(22)00031-0 (PMC9021027; doi:10.1016/S2589-7500(22)00031-0)
Supplement: Supplementary appendix [file mmc1.pdf]

THE LANCET
Digital Health

Supplementary appendix

This appendix formed part of the original submission and has been peer reviewed. We post it as supplied by the authors.

Supplement to: Sin J, Henderson C, Elkes J, et al. Effect of digital psychoeducation and peer support on the mental health of family carers supporting individuals with psychosis in England (COPe-support): a randomised clinical trial. *Lancet Digit Health* 2022; published online April 1. https://doi.org/10.1016/S2589-7500(22)00031-0.

Study Protocol

EFFIP (E-support for Families and Friends of Individuals affected by Psychosis): A randomised controlled trial of a co-produced online intervention for carers
(Short title – RCT of COPe-support online resource for carers)

Sponsor's Project Number: 18.0027
REC Reference Number: 18/SC/0104
IRAS ID: 240005
Protocol Version and Date: v1.2 dated 27.02.2018
Trial Registration for Current Controlled Trials unique registration number: TBC

CHIEF INVESTIGATOR (CI):
Dr Jacqueline Sin
NIHR Post Doctoral Research Fellow
Population Health Research Institute
St George's, University of London
Cranmer Terrace
London
SW17 0RE
Phone: 02087255530 & 07817027035
Email: jasin@sgul.ac.uk

SPONSOR: St George's, University of London

SPONSOR REPRESENTATIVE:
Dr Deborah McCartney
Joint Research & Enterprise Office
St George's, University of London and St George's Healthcare NHS Trust
Cranmer Terrace
London
SW17 ORE
Phone: 02087250892
Email: dmccartn@sgul.ac.uk
Fax: 02087250794

Funding Source: National Institute for Health research (NIHR) Post doctoral research fellowship awarded to Dr Jacqueline Sin (Reference: PDF-2015-08-035)

Information in this protocol is confidential and should not be disclosed, other than to those directly involved in the execution or the ethical review of the study, without written authorisation from St George's Joint Research & Enterprise Office (JREO) or its affiliates.

Statement

The Chief Investigator (CI) and the Sponsor representative have discussed this protocol version. The investigators agree to perform the investigations and to abide by this protocol except where departures from it are mutually agreed in writing.

The Investigator agrees to conduct the trial in compliance with the protocol, GCP, the Data Protection Act (1998), the Trust Information Governance Policy (or other local equivalent), the Research Governance Framework (2005 2nd Edition), the Sponsor's SOPs, and other regulatory requirements as appropriate.

This protocol has been written in accordance to the Sponsor's procedure identified as: JREOSOP0039 "Protocol Design" and is intended for use at UK sites only.

**Chief Investigator**<br>Dr Jacqueline Sin<br>NIHR Post Doctoral Research Fellow<br>St George's, University of London	Signature<br>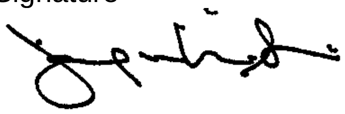	Date<br><br>26.01.2018

Acknowledgements and Protocol contributories

Dr Jacqueline Sin (JS) (St George's, University of London, Cranmer Terrace, London SW17 0RE) conceived the study; JS initiated the study design of the overall EFFIP study, including the randomised controlled trial (RCT) on an online intervention which is specified in this protocol. JS sought and received the NIHR award for the conduct of the EFFIP study including the RCT. JS receives academic supervision from Dr Steve Gillard (of St George's, University of London) and Dr Claire Henderson (of King's College London) in the design of the study, including this study protocol. JS is the sole author of this protocol but has consulted Dr Gillard and Dr Henderson, the Trial Statisticians (Dr Victoria Cornelius and Dr Tao Chen) and the Chair of the Trial Steering Committee in finalising this protocol.

This study, as part of an overall bigger study entitled "EFFIP (E-support for Families and Friends of Individuals affected by Psychosis): A randomised controlled trial of a co-produced online intervention for carers", is funded by the National Institute for Health Research (NIHR) (awarded to Dr Jacqueline Sin through its Post Doctoral Research Fellowship, PDF-2015-08-035). The views expressed in this document are those of the author(s) and not necessarily those of the NHS, the NIHR or the Department of Health.

Contents

1 List of abbreviations 5
2 Roles and Responsibilities 6
Principal Investigator (PI): Dr Jacqueline Sin 6
3 Study synopsis 8
4 Background 10
4.1 Study Rationale 11
5 Study aims and objectives 11
5.1 Primary objective 11
5.2 Secondary objective 11
5.3 Hypothesis 11
6 Methods 11
6.1 Trial design 12
6.2 Intervention plan and rationale 13
6.3 Additional treatment/interventions 14
6.4 Schematic of study design 14
7 Participation selection criteria 15
7.1 Inclusion criteria 15
7.2 Exclusion criteria 16
8 Participant recruitment process 16
9 Study procedures 17
9.1 Eligibility screening, informed consent and enrolment 17
9.2 Randomisation procedure 18
9.3 Allocation concealment and blinding 18
9.4 Discontinuation/withdrawal of participants and stopping rules 18
9.5 Participant transfer 19
9.6 Lost to follow up 19
9.7 Definition of the end of trial 19
10 Study and data collection procedures 20
10.1 Outcome measures 21
10.2 Screening assessments 21
10.3 Baseline assessments 21
10.4 Subsequent assessments 21
10.5 Summary flow chart of study assessments 21
10.6 Methods 23
10.6.1 Laboratory procedures 23
10.6.2 Radiology or any other procedure(s) 23
11 Safety events 23
11.1 Definitions 23
11.2 Recording AE/SAE/UC 24
11.3 Investigator responsibilities relating to safety reporting 24
11.4 Notification of deaths 25
12 Data management and quality assurance 25
12.1 Confidentiality 25
12.2 Data collection mechanisms 25
12.3 Incidental Findings 26
12.4 Data handling and analysis 26
13 Archiving arrangements 27

14 Statistical design 27
14.1 Statistical input in trial design 27
14.2 Endpoints 27
14.2.1 Primary endpoints 27
14.2.2 Secondary endpoints 28
14.3 Sample size and recruitment 28
14.3.1 Sample size calculation 28
14.3.2 Planned recruitment rate 28
14.4 Statistical analysis plan 29
14.4.1 Summary of baseline data and flow of patients 29
14.4.2 Primary endpoint analysis 29
14.4.3 Secondary endpoint analysis 30
14.4.4 Sensitivity and other planned analyses (if applicable) 30
14.5 Interim analysis 30
Not applicable. 30
14.6 Other statistical considerations 30
15 Committees involved in the trial 31
16 Direct access to source data 31
17 Ethics and Research Governance requirements 31
17.1 Annual Progress Reports (APRs) 31
17.2 Notification of serious breaches of GCP and/or the protocol 31
18 Finance 32
19 Insurance and indemnity 32
20 IP and development policy 32
21 Publication policy 32
21.1 Before the official completion of the Trial 33
21.2 Up to 180 days after the official completion of the Trial 33
21.3 Beyond 180 days after the official completion of the Trial 33
22 Statement of Compliance 34
23 List of Protocol appendices 34
24 References 35
Appendix 1 – TSC terms of reference 38

1 List of abbreviations

AE	Adverse Event
CI	Chief Investigator
CRF	Case Report Form
CTU	Clinical Trials Unit
COPe-support	Carers fOr People with Psychosis e-support resource
EFFIP	E-support for Families & Friends of Individuals affected by psychosis
GCP	Good Clinical Practice
HRA	Health Research Authority
ICF	Informed Consent Form
ISF	Investigator Site File
NHS R&D	National Health Service Research & Development
PI	Principal Investigator
PIS	Participant Information Sheet
PRG	Project Reference Group
RCT	Randomised Control Trial
REC	Research Ethics Committee
SAE	Serious Adverse Event
SAP	Statistical Analysis Plan
SDV	Source Document Verification
SOP	Standard Operating Procedure
SSA	Site Specific Assessment
TSC	Trial Steering Committee
UC	Unintended Consequences

2 Roles and Responsibilities

Principal Investigator (PI): Dr Jacqueline Sin
NIHR Post Doctoral Research Fellow
St George's, University of London
&
Honorary Consultant Nurse
South West London and St George's Mental Health NHS Trust
E-mail: jasin@sgul.ac.uk
Phone: 02087255530 & 07817027035

Collaborator: Dr Claire Henderson
Clinical Senior Lecturer in Psychiatry
Department of Health Services & Population Research
Institute of Psychiatry, Psychology & Neuroscience, King's College London
De Crespigny Park, London SE5 8AF
E-mail: claire.1.henderson@kcl.ac.uk

Collaborator: Dr Steve Gillard
Reader, Social & Community Mental Health
Population Health Research Institute
St George's, University of London
Cranmer Terrace, London SW17 0RE
E-mail: sgillard@sgul.ac.uk

Statistician: Dr Victoria Cornelius
Senior Lecturer in Clinical Trials
Deputy Head of Statistics
Imperial Clinical Trials Unit, School of Public Health, Imperial College London
Stadium House, 68 Wood Lane, London W12 7RH
E-mail: v.cornelius@imperial.ac.uk

Statistician: Dr Tao Chen
Medical Statistician
Department of Clinical Sciences, Liverpool School of Tropical Medicine
Pembroke Place, Liverpool L3 5QA
E-mail: Tao.Chen@lstmed.ac.uk

Trial Steering Committee:
(The TSC will concentrate on the progress of the trial in relation to protocol compliance and review of any participant safety considerations and to advise the sponsor and/or CI of any decisions. See Appendix 1 for Terms of Reference of TSC)

Chair: Dr Bryn Lloyd-Evans
Senior Lecturer in Mental Health and Social Care
Division of Psychiatry, UCL
6th Floor, Maple House, 149 Tottenham Court Road, London W1T 7NF
Email: b.lloyd-evans@ucl.ac.uk

Independent Statistician: TBC

Subject-expert member: Professor Jo Smith
Professor of Early Intervention & Psychosis

Institute of Health & Society
University of Worcester
Email: j_smith@worc.ac.uk

Carer member 1: Christine Lewis

Carer member 2: Evadine Okoye

Carer member 3: Paul Kenny

CI/PI: Dr Jacqueline Sin

Two of the study team members (maximum): Dr Claire Henderson; Dr Steve Gillard; Dr Victoria Cornelius; Dr Tao Chen; Luke Woodham (eLearning expert); or Aurora Sese (eLearning expert).

3 Study synopsis

**Brief title:**	RCT of COPe-support online resource for carers
**Official title:**	EFFIP (E-support for Families and Friends of Individuals affected by Psychosis): A randomised controlled trial of a co-produced online intervention for carers
**Sponsor reference number:**	18.0027
**Public database identifier**	ISRCTN Reference - TBC
**Study type & Phase**	Effectiveness evaluation study
**Study design**	Randomised controlled trial commencing with an internal pilot RCT to evaluate the effectiveness of an online intervention to promote carers' wellbeing.
**Study Population/disease condition**	Informal or family carers supporting a loved one affected by psychosis.
**Eligibility criteria:**	**Inclusion criteria:**<br>Informal or family carers (carers thereafter) supporting a loved one affected by psychosis. Carers include family members with biological or non-biological relationship or a close friend supporting an individual with whom they have an emotional bond, over a sustained period of time, on an unpaid basis. Plus the following inclusion criteria:<br>▪ Carers need to have at least weekly contacts with the cared-for person, although these contacts could be in a variety of formats, e.g. face to face, phone calls, emails, or social media such as facebook, twitter, text messages;<br>▪ Adult aged 18 or over;<br>▪ Are living in England during the study period;<br>▪ Able to communicate in English in usual online communications;<br>▪ Have daily access to the internet.
	**Exclusion criteria:**<br>The exclusion criteria are:<br>▪ Those aged below 18;<br>▪ Those who cannot communicate in English; and<br>▪ Those not able to access and use online communications.
**Target number of participants**	360
**Criteria for evaluation**	**Primary outcome measure(s):**<br>Carers' mental wellbeing measured using the Warwick-Edinburgh Mental Wellbeing Scale (WEMWS) at 20 weeks (end of intervention)
	**Secondary outcome measure(s)**
	▪ Carers' mental health knowledge using Mental Health Knowledge Schedule (MAKS)<br>▪ Carers' experience of caregiving using Experience of Caregiving

	Inventory (ECI)<br>▪ Carer's quality of life using EQ-5D-5L<br>▪ Carer's perceived social support using Carer Wellbeing & Support Questionnaire (CWS)<br>▪ Carer's satisfaction with the intervention as a process evaluation outcome with post-use individual interview<br>▪ Adverse psychological effects to carers
**Sources of funding**	National Institute for Health Research Post Doctoral Research Fellowship
**Anticipated start date:**	05.03.2018
**Anticipated primary completion date:**	31.12.2020
**Sponsor/Co-Sponsor**	St George's, University of London
**Key Contact names**	Sponsor representative: Dr Deborah McCartney<br>Email: dmccartn@sgul.ac.uk<br>Tel: 02087250892<br><br>Chief Investigator: Dr Jacqueline Sin<br>Email: jasin@sgul.ac.uk<br>Tel: 02087255530 / 07817027035

4 Background

It is estimated that approximately 1.5 million people in the UK are caring for a family member or friend with a mental illness.(1,2) Of these illnesses, psychotic disorders such as schizophrenia, are recognised as among the most common and severe.(3,4) Psychosis costs society £11.8 billion a year and it is widely recognised that coping with psychosis is often challenging and difficult not just for the individuals themselves but everyone close to them. The importance of relatives and friends in mental health care (commonly referred to as informal carers or carers as referred to thereafter) is well established, in that individuals in receipt of support and care from their informal caregiving networks have a better prognosis and enhanced quality of life.(5,7,8) However, the burden of caring often means that carers of people with psychosis experience high levels of distress and increased vulnerability to depression and anxiety.(3,4) Carers who lack support and resources to cope are less likely to engage in caring for their loved ones, and more likely to exhibit critical or hostile behaviour towards the cared-for, albeit unintentionally, which in turn jeopardises the individual's clinical outcomes and wellbeing.(3-5) Effective interventions for maintaining and promoting the mental wellbeing of carers are needed and indeed now part of the legal requirement of the Care Act.(9)

Enhanced psychoeducation (that is information giving on the illness and coping strategies for related caring issues)(1,5) that targets carers of individuals affected by psychosis, has a strong evidence-base for its effectiveness in enhancing carers' knowledge and coping with their caring roles, which in turn, impacts positively on the cared-for persons' recovery in terms of reduced relapse and improved social functioning.(7,8,10,11) However, despite the established effectiveness of psychoeducation and the NICE recommendation of its wide-spread implementation,(1,5) carers are rarely provided with such intervention in routine mental health and social care services. In addition to the pitfall in implementing evidence-based interventions due to various factors such as lack of funding, workforce competency and service priorities centred upon the service users (or patients), carers have also expressed their need for such support to be delivered to them as a flexible, robust and self-paced package, ideally through an online medium so to suit their busy lifestyle and many other commitments in addition to caregiving.(3,4,12-14) In addition to information and advice given by mental health professionals, carers also identified peer-to-peer support as particularly useful in reducing their sense of isolation.(15-16) There have been a handful of research studies showing promising benefits of ehealth interventions targeting carers, covering a range of long-term and severe diseases including diabetes, dementia and cancer.(17-18) Recent research indicates that the internet can be a promising medium for providing such a resource for carers of individuals affected by psychosis.(19-23)

The EFFIP (E-support for Families & Friends of Individuals affected by Psychosis) Project: A randomised controlled trial of a co-produced online intervention for carers, aims to develop and evaluate an online resource to promote the wellbeing and caregiving experiences of carers supporting a loved one with psychosis.(24) The overall EFFIP study lasts for 5 years (from 2016) and uses a mixed-method approach combining qualitative, quantitative, usability-testing and randomised controlled trial with inbuilt process evaluation methods, along the development, feasibility and evaluation phases of the Medical Research Council (MRC) framework for complex interventions.(25) From March 2018, the EFFIP project progresses into the evaluation phase of the MRC framework (25), to evaluative the clinical effectiveness of our online intervention in improving carers' wellbeing and other health-

related outcomes. Ethical and research governance approval are being sought for the evaluative study specifically.

4.1 Study Rationale

We have successfully undertaken five studies in the first two years - the development and feasibility/piloting phases - of the EFFIP project (2016-2017).(24) We have meta-synthesised findings from systematic reviews and ideas collected from carers and service users through qualitative studies to inform the development of the online intervention.(26) The intervention-prototype, called COPe-support (an acronym for our online intervention which stands for Carers fOr People with Psychosis e-support) has been further refined through an iterative consultation process with carers and extensive Public and Patient Involvement (PPI) activities along the build process.(24) We have also established usability and feasibility of delivering COPe-support online through a mixed-method usability evaluation study recently.(27) Hence, the EFFIP project is on target to progress to its third and last phase: evaluation of the intervention effectiveness.(25) This current protocol is for a randomised controlled trial including an internal pilot to evaluate the clinical-effectiveness of COPe-support in promoting carers' wellbeing and other outcomes.

5 Study aims and objectives

The aim of the EFFIP project is to evaluate an internet-based multi-component support intervention for carers of individuals affected by psychosis, in promoting carers' mental wellbeing with a focus on helping them to gain essential knowledge and coping strategies to support the service users in their caring role.

5.1 Primary objective

The current study has specific objectives, as follows:

1. To evaluate the clinical effectiveness of the intervention in improving carers' mental wellbeing (measured using the Warwick-Edinburgh Mental Wellbeing Scale (WEMWS, 28) at 20 weeks (end of intervention).

5.2 Secondary objective

1. To determine the clinical effectiveness of the intervention in improving carers' appraisal of caregiving experiences and other health-related outcomes.
2. To determine the acceptability of the online intervention as perceived by the carers.
3. To determine the intervention usage by all participants allocated to the active intervention arm, to establish usage pattern and to correlate effectiveness with usage.

5.3 Hypothesis

The primary hypothesis is that the online intervention, COPe-support, is superior to a waitlist control with respect to improving carers' wellbeing, measured using WEMWBS (28) at the end of intervention use (i.e. 20 weeks).

6 Methods

6.1 Trial design

The evaluative study is a RCT with two stages: an internal pilot RCT; and a full RCT. The RCT uses a two-arm, individually randomised controlled superiority trial design comparing the online intervention (in addition to usual care) with a waitlist control (in addition to usual care). Both the study procedures and the intervention will be delivered completely online. Participant pathway through the RCT is shown in the CONSORT diagram (29) in Section 6.4.

Internal pilot

The study will include a 12-month internal pilot to assess to criteria: recruitment rate and retention rate of the intervention.

1) Recruitment rate
   - If recruitment rate is < 50% of the planned rate then the criteria is not met.
   - If the recruitment is between ≥ 50% but < 80% then the criteria is partially met.
   - If the recruitment rate is ≥ 80% then the criteria is met.
2) Retention rate
   - If the retention rate is <50% of the planned rate then criteria is not met.
   - If the retention is between ≥50% but <80% then the criteria is partically met.
   - If the retention rate is ≥80% then the criteria is met.
3) Usage
   - If there is valid usage data for <50% of participants, then the criteria is not met.
   - If there is valid usage data for ≥50% but <80% of participants, then the criteria is partically met.
   - If there is valid usage data for ≥ 80% of participants, then the criteria is met.

If all criteria are not fully met, the TSC will review strategies to improve recruitment, retention and usability.

The internal pilot RCT will run for the initial 12 months (March 2018 – February 2019) and aims to recruit one-third of the total study participants.

The main trial

If the internal pilot criteria are met the main trial will continue and run for 18 months (March 2019 – September 2020) in which the remaining two-third of the study participants will be recruited and assessed.

Process evaluation

A process evaluation study involves both the internal pilot and main trial.

The process evaluation will involve:
(1) Collection of usage data, e.g. number and frequency of log-ons, time spent online, components visited, by all participants allocated to the intervention arm. These data will be monitored to establish usage rate and to correlate effectiveness with usage.
(2) Interviewing a purposive sample of 20% of the participants after they complete using the EFFIP intervention and all the data collection procedures (i.e. n ~ 36 out of 180 depending on data saturation). Purposive sampling will be used to identify the participants to ensure representation of diverse experiences and views from those with ethnic minority backgrounds, frequent and infrequent users whose experience may impact on their usage and outcomes. In addition to seek to understand carers' experiences and perceived acceptability of the intervention, these qualitative findings will provide invaluable insights into planning wider implementation with relevant engagement and facilitation consideration.

6.2 Intervention plan and rationale

Participants can access the intervention online platform (either for the COPe-support or the non-interactive resources information webpage) 24/7 throughout the study (20 weeks) and the follow-up (a further 20 weeks) duration. The intervention is designed for participants' use at their own home or base, through the internet. Both the intervention and the control platforms provide a direct web link for participants for either emotional (e.g. for those who want direct contact from the online facilitator for support or queries) or technical (e.g. for those who has difficulties navigating the site) support. We encourage the participants to spend about an hour per week during the intervention phase (i.e. 20 weeks) to go through the content, in an order which best suits their needs and interest. Alongside using the intervention, participants continue with their usual care which in most cases include access to local carer support groups or personnel (statutory or non-statutory), their own GP and primary care service.

Development and co-production of the intervention

The development of the online multi-component psychoeducational intervention is informed by the previous phases of the overall project, i.e. the theoretical development and modelling/feasibility phases in the first two years.(25) The theoretical phase comprises two stages, including two systematic reviews (10,30) and a focus group study with service users with lived experience of psychosis and family carers.(26) One systematic review investigates the effectiveness of psychoeducational interventions using any delivery methods on carers' wellbeing and health morbidities and also the correlation of intervention duration and dosage with effectiveness.(10) The second review focuses on scoping eHealth interventions targeting family carers of people with long term illness.(30) Through this review, we investigated the common information communication and technology (ICT) features and implementation considerations used in such interventions. We meta-synthesised findings from these three studies to inform the design and content of our intervention. Our intervention, COPe-support is based upon the stress-appraisal and coping theory (31) that is commonly used in conventional psychoeducational interventions targeting family members and relatives.

The second - modelling/feasibility – phase of the EFFIP project saw the establishment of the Expert Advisory Group (EAG) which comprised key stakeholders including carers, individuals with lived experience of psychosis, clinicians working in mental health and primary care settings, health services researchers, mental health charity representative and e-learning experts. The EAG oversaw the development and build process of the online intervention, through a series of consultation forums. Along the build process, the iterative consultations with EAG was further supplemented by a focus group study with carers to

collect feedback on the developing drafts of the intervention. At the end of the modelling/feasibility phase, we conducted a usability study to evaluate the usability and feasibility of the intervention-prototype.(27) The usability study comprised three sub-studies combining remote usability test and think-aloud sessions with carers and heuristic evaluation with e-learning experts. The final draft of the intervention was further refined based on the results of the usability study.

Design and content of the intervention

We report the intervention design according to the TIDieR guideline.(32) The online intervention is called COPe-support, an acronym of "Carers fOr People with Psychosis e-support resource". It is built and hosted by CANVAS, a Massive Open Online Course (MOOC) platform (33) but access to the intervention and its control condition is limited to consented participants only during the study period. COPe-support has multiple components and the following elements: information on psychosis; common treatment and caring strategies for symptoms; information focusing on self-care strategies for carers; a peer support element that uses a virtual discussion forum and blog space between participants to share experiences and discuss commonly encountered issues; a "Ask the Experts" forum where participants can post questions to an expert panel comprising health and social care professionals and campaigners; and a "Further resources" section with supplementary web links to relevant external resources. Altogether, the content is grouped into 12 sections: a home page with introduction and navigation videos; eight information-focused sections; two online forums; and a "Further resources" unit. Throughout the intervention, there are cognitive-behavioural orientated exercises and reflection points designed to encourage participants to take stock of self-care and caregiving skills and integrate those into their own life. The content contains a mixture of textual and audio-visual information devised by the study team and contributed by experts through experience (both ex- and current service users who have personal experience of psychosis, and carers) and clinical-academic experts. An online facilitator, a mental health nurse with over 20 years' experience specialising in psychosocial interventions for people with psychosis and their family carers, monitors and moderates the online intervention daily during the week. She also posts weekly updates within the intervention online news forum to all participants with an aim to keep them engaged. The intervention can be accessed through computer (desktop or laptop) as well as mobile devices (e.g. tablets and mobile phones).

Control condition

The control condition is a non-interactive resource website providing information and multiple web links to various external resources/services, accessed through the same CANVAS platform, i.e. the stand-alone "further resources" unit. Resources covered include voluntary and statutory services, books, online resources, information sources relating to psychosis and caregiving. After the follow up data collection, participants allocated to the control condition will be given access to the COPe-support.

6.3 Additional treatment/interventions

Not applicable.

6.4 Schematic of study design

See Figure I below.

Figure I – CONSORT diagram for EFFIP RCT

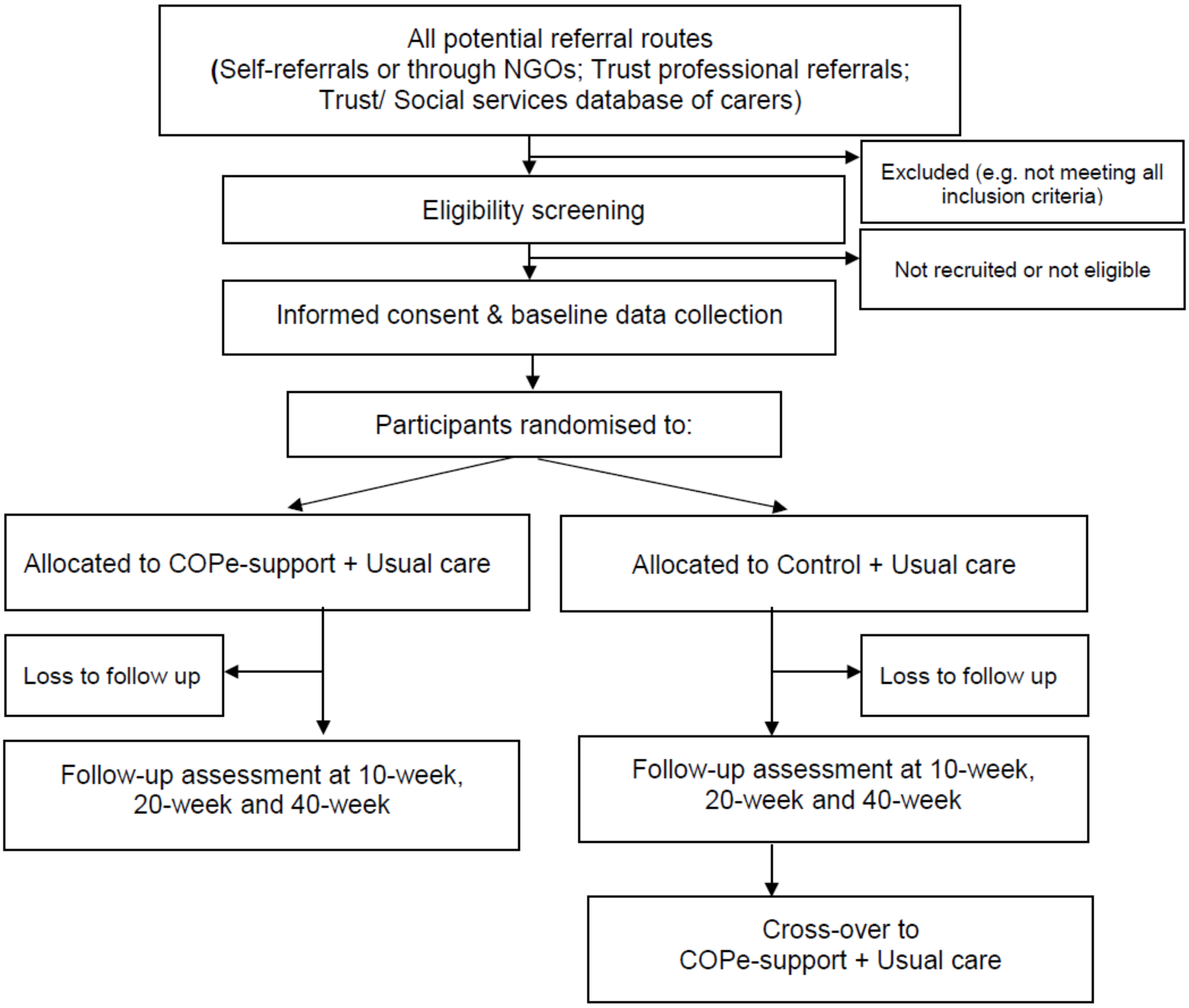

7 Participation selection criteria

The online intervention, COPe-support is designed for carers supporting a loved one affected by psychosis. Carers include family members with a biological or non-biological relationship (e.g. parents, siblings, spouses, and other relatives) or a close friend supporting a loved one affected by psychosis. Only one carer per psychosis patient will be included in the study.

7.1 Inclusion criteria

Carers who are:

- Adult aged 18 or over;
- Those who have at least weekly contacts with the cared-for person, although these contacts could be in a variety of formats, e.g. face to face, phone calls, emails, or social media such as facebook, twitter, text messages;
- Living in England during the study period;

- Able to communicate in English in usual online communications; and
- Have daily access to the internet.

7.2 Exclusion criteria

Regrettably, we cannot accept carers with the following characteristics into the RCT:

- Those aged below 18;
- Those who cannot communicate in English;
- Those not able to access and use online communications; and
- Those who cares for a loved one affected by psychosis but another relative/close friend who also shares a caring role for the same individual has already participated in the study (to avoid a clustering effect).

8 Participant recruitment process

Recruitment is scheduled from March 2018 to February 2020. Recruitment for the internal pilot will take place over the initial 12 months (February 2018 – January/February 2019), followed by another 18-month period for the full RCT.

Participant recruitment at a site will only commence once evidence of the following approval/essential documents are in place:

1. REC and HRA approval; and
2. Final sponsorship and host site permission.

We plan to recruit carers through the following strategies:

1. Through our project website (http://cope-support.org) and associated social media strategy (using mainly Twitter) and engaging with relevant organisation social media agencies (such as voluntary organisations, local carer organisations);
2. Mental Health NHS Trusts; and
3. Voluntary/non-governmental agencies providing support for carers (such as SANE, Carers UK, Rethink Mental Illness, Carers Trust and Mind).

We will work closely with the Clinical Research Network (CRN) to optimise reach of recruitment activities in all the recruitment sources/sites. We also plan additional strategies to optimise recruitment if required. These include: a planned ‘lead-in’ period of three months prior to each intervention-cohort start-time (total six cohorts over the trial period), during which active recruitment activities will be undertaken and eligible participants can enrolment onto the study through our website; a scheme of goodwill payments for participants; using online recruitment strategies to target those already using the internet to seek help; and further active identification and approach strategies.

As part of our standard recruitment strategies, we plan to identify carers from our recruitment sources (i.e. NHS mental health services and voluntary organisations). We will only contact carers who are already in direct contact with each service about our study. In some NHS services (such as Community Mental Health Team, Early Intervention for Psychosis Service, Patient Advice & Liaison Services (PALS), and Recovery Colleges), there are databases/lists of carers who are currently or have previously attending/attended support groups, information-giving sessions, consultation forums, and/or receiving input from clinical staff and support workers. In voluntary organisations (such as Rethink Mental Illness, Carers’ Trusts), there are often local carers support groups and communication network/database (via email or post) run by volunteers. We plan to identify and invite carers to take part in this study through email and/or letters, using such pre-existing carers

databases in NHS and voluntary organisations, with the support of the NHS-employed CRN workers and/or clinicians/support workers in each site.

In addition, our researchers (the CI, PI and CRN staff) will make presentations about the study to NHS staff and provide them with study information. We will ask the staff to help pass on information about the study to carers. We will undertake the same publicity and advertising procedures with carers/ families at carers' services (such as carers groups, Recovery Colleges, voluntary sector activities). Presentations will be made on site and at a time convenient for the services/staff by our research staff and each presentation will not take more than 10 minutes of staff time at any one time. Carers will be encouraged to contact the research team if they would like to have further information about the study. Alternatively, we will also encourage the staff to check with the carers if they would like the research team members to contact them with their permission sought for their name and contact details to be passed on.

Furthermore, we have also planned contingency recruitment strategies in the event of slow or below expectation recruitment rate. These include: recruiting carers through service users who use NHS services, subject to service users and their care coordinator/consultant's agreement; expanding recruitment sites; and extending recruitment time frame. In the event of recruiting carers through service users, we plan to send out study flyer and an invitation letter to the service users who are open to NHS service with a known diagnosis of psychosis and ask for their help to pass on the study information to their carer.

9 Study procedures

9.1 Eligibility screening, informed consent and enrolment

Potentially eligible carers are encouraged to contact the researcher or the local research supporting staff (such as CSO/CSA or Principal Investigator, PI) and/or visit our project website where detailed information about the study is shown (and also downloadable). Potential participants can also make contact with the study team through a direct email link on our study website for any outstanding queries.

Interested participants will be prompted to read through our Participant Information Sheet (PIS) and Ground Rules before going through an online eligibility check procedure by way of completing an online checklist of all inclusion and exclusion criteria. Eligible participants will be invited to give informed consent online. Non-eligible carers are invited to leave their contact details if they would like to have the eligibility criteria further clarified and/or be signposted to other available resources.

Informed consent will be obtained via our secure online study platform from each participant. Assistance from the research team staff will be provided to the participants through online, phone or face to face media, as required and as preferred by the participant. As part of the informed consent procedure, we ask the participant to provide their full name, a valid email account and a mobile phone number which we will use later to send them their login details for accessing the interventions.

Consented participants will then be invited to give information on their socio-demographics and their caregiving situation as part of the enrolment process. Data collected at enrolment include gender, age, ethnicity, employment/education status, marital status, living arrangement (with the cared-for person or not), relationship with the cared-for person, and time and role the carers commit to their caring role, the cared-for individual's age, gender and diagnosis, length of time since they first became unwell.

9.2 Randomisation procedure

As there is a lead-in period of three months prior to the intervention (-cohort) start time, and during this period, eligible participants can enrol and consent to join the RCT freely and be informed of the intervention start time. Nonetheless, consented and enrolled participants will only be invited to complete baseline assessment within a two-week window of the intervention start time.

Only consented participants who have completed baseline assessment will be entered into the randomisation process. Carer-participants are randomised on an individual level with participants treated as independent units of randomisation. Participants will be allocated into the two arms (i.e. either COPe-support + usual care or Control + usual care)
using randomised permuted blocks stratified by gender via an online bespoke system, developed and hosted by the King's CTU (http://www.ctu.co.uk).

The online facilitator (JS) will submit participants' information (i.e. initials, date of birth and study Id number) for randomisation individually, as soon as the participants complete baseline assessment.

9.3 Allocation concealment and blinding

Allocation to intervention groups will be centrally assigned by the online randomisation process. The online facilitator or the eLearning experts will enrol participants as allocated by the randomisation system. The Trial Statisticians will perform a check on the allocations at the end of the internal pilot (and the full RCT) to ensure the allocation to groups are done according to the sequence. The facilitator and participants will know intervention allocation and hence neither will be blinded for the study. All outcome data are self-reports and input directly online by participants. Purposeful selection and invitation of participants for the process evaluation study will only be initiated after their completion of follow up data collection.

After all the outcome measure data and usage data are collected, the CI (JS) will take responsibility in cleaning and checking the data, before transferring the data onto SPSS or Stata for analysis. The analyst will be sub-group blind when undertaking the analysis. A detailed statistical analysis plan will be written which will specify the analysis to be undertaken prior to any data being extracted and examined from the database. Unblinding will only be initiated after the initial data analysis are completed.

9.4 Discontinuation/withdrawal of participants and stopping rules

Withdrawal of consent from the participants

As participation in the trial is entirely voluntary, the participant may choose to discontinue participation at any time without penalties or loss of benefits to which they may be entitled. Although not obliged to give a reason for discontinuing their participation a reasonable effort will be made by the study team to establish this reason, whilst remaining fully respectful of the participant's rights.

Withdrawal of participants by the study team

In the unlikely circumstances, stopping rules including discontinuation or withdrawal of participants will be considered by the core study team, in consultation with the TSC, subject to identification of serious adverse events including serious breach of ground rules (e.g. a participant posting offensive message that jeopardise their own and others' confidentiality) which are deemed to be study-related. See Section 11.

The follow-up for participants withdrawn from the study

If a participant chooses to discontinue or be withdrawn from the study (initiated by themselves or by the study team), they will be continued to be followed up as closely as possible to the follow-up schedule defined in the protocol with a particular priority on the 20-week outcomes, providing they are willing. However, if the participant confirms they do not wish to participate in the scheduled follow-up data collection time points then data that has already been collected will be kept and analysed according to the ITT principle for all participants. The study team will use such data confidentially in connection with the purposes for which consent is being sought. These considerations and arrangement will be clearly explained to the participants in the PIS.

9.5 Participant transfer

Not applicable.

9.6 Lost to follow up

To optimise data collection at all time points, we have devised the following strategies:

- We give details in advance of the intervention start time as well as the follow-up time points, for the participants' attention;
- We send weekly email updates through our CANVAS platform with an intention to keep participants engaged throughout the study period;
- Only those participants who have completed baseline assessment (performed at most 2 weeks prior to start of intervention) will be randomised into the groups;
- At each time point, we will send up to 4 emails and text messages, at a weekly interval, to invite and remind participants to complete data collection through a direct weblink which is accessible 24/7;
- We will offer participants a goodwill payment after they complete each round of data collection (i.e. £10 for baseline assessment, £5 for mid and end of intervention assessment respectively, and £10 for 40-week follow-up assessment);
- Participants allocated to the control group will access the "Further resources" (+ usual care) through our CANVAS platform, and will be sent weekly updates and reminders for data collection via email/text with a direct weblink to the data-collection site; and
- Control group participants will be able to access the COPe-support resource after they complete all follow-ups.

9.7 Definition of the end of trial

The trial will end by 31st December 2020 when all the data collection procedures have been completed.

10 Study and data collection procedures

The study data collection procedures described here apply to both the internal pilot and full RCT. Figure II shows all the outcome measures and the schedule at which they are completed.

Figure II – Schedule of data collection

Time point		STUDY PERIOD					
		Enrolment	Allocation	Post-allocation			Process evaluation
		T-1	T0	T1	T2	T3	T4
		Wk -18-0	Wk 0-2	Wk 10	Wk 20	Wk 40	Wk 41-45
ENROLMENT	Eligibility screening	X					
	Informed consent	X					
	Sociodemographic & caring data	X					
	Randomisation		X				
INTERVENTIONS	COPe-support			X	X	X	
	Waitlist			X	X	X	
ASSESSMENTS	Caring-related data (including health & social service use)		X	X	X	X	
	WEMWBS		X	X	X	X	
	MAKS		X	X	X	X	
	ECI		X	X	X	X	
	EQ-5D-5L		X	X	X	X	
	FQ		X	X	X	X	
	CWS		X	X	X	X	
	Perceived acceptability						X
	Usage data						X

T-1: Pre-randomisation; T0: baseline and randomisation; T1: mid-intervention; T2: end of intervention; T3: 40-week follow up; T4: after follow-up outcome data collection; WEMWBS: Warwick-Edinburgh Mental Wellbeing Scale; MAKS: Mental Health Knowledge Schedule; ECI: Experience of Caregiving Inventory; EQ-5D-5L: EuroQol 5 level version of EQ-5D; FQ: Family Questionnaire; CWS: Carer Wellbeing & Support Questionnaire.

10.1 Outcome measures

The primary outcome is carers' mental wellbeing, assessed using Warwick-Edinburgh Mental Wellbeing Scale (WEMWBS) (28), at end of intervention use (i.e. 20 weeks).

Secondary outcomes include the following domains, assessed using the respective tools:

- Carer's mental health knowledge, assessed using Mental Health Knowledge Schedule (MAKS) (34);
- Carer's caregiving experiences, both negative appraisal and positive appraisal, measured by subtotals of Experience of Caregiving Inventory (ECI) (35);
- Family relationship and communication, as assessed by Family Questionnaire (FQ) (36);
- Carer's perceived social support, measured with Carer Wellbeing and Support Questionnaire (CWS) (37); and
- Quality of Life assessed using EuroQoL 5-level EQ-5D (EQ-5D-5L) (38).

In addition, in the process evaluation study, we will conduct individual interviews to collect qualitative data from a purposive sample of participants for their experience and perceived acceptability of the online intervention. We will also extract usage data for all participants allocated to the intervention group.

10.2 Screening assessments

Procedures and content of screening and sociodemographic assessments are reported in Section 9.1 Eligibility screening, informed consent and enrolment.

10.3 Baseline assessments

Baseline assessment will be conducted within a maximum of 2-week duration of the intervention start time (i.e. maximum 2 weeks before or 2 weeks after the intervention starts). Consented participants will be invited to give data on recent (within the last 2 weeks) caregiving-related factors (e.g. has the cared-for person had a relapse or in need of crisis or home treatment service) and all the outcome measures (listed in Section 10.1). All assessments are completed online through our secured online platform, which allows direct data input by participants. Only after the participants have completed the baseline assessments, their details will be entered into the online randomisation system for allocation.

10.4 Subsequent assessments

There are three further time points after randomisation for outcome data collection: at 10 weeks (mid-intervention use); 20 weeks (end of intervention-use); and 40 weeks (20-week follow up post intervention). At each of these assessments, the participants will be invited to complete the caregiving-related factors data and all the primary and secondary outcome measures, as aforementioned.

The process evaluation is embedded within the RCT and will collect usage and qualitative data after the completion of all outcome data (i.e. post week 40).

10.5 Summary flow chart of study assessments

The schedule of assessments, together with that of the study procedures, is presented in Figure III.

Figure III – Schedule of study procedures and assessments

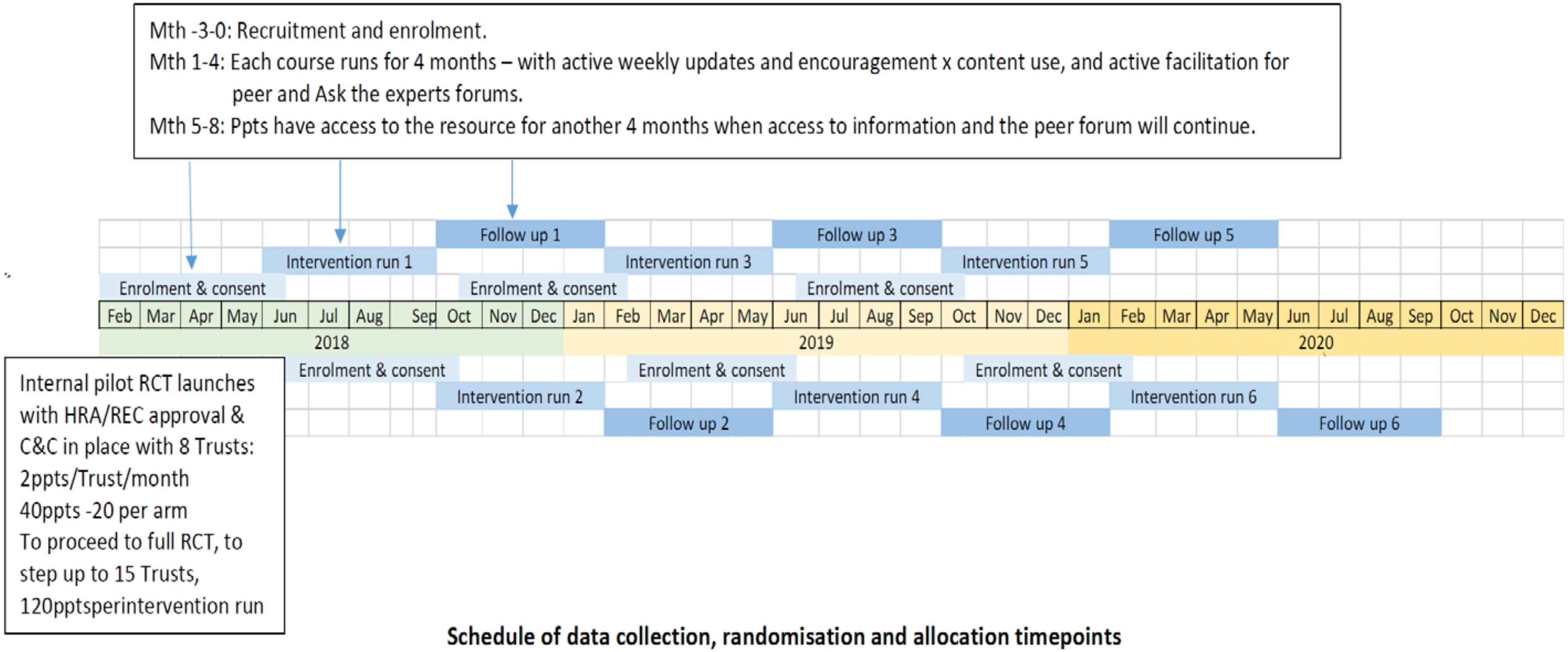

Schedule of data collection, randomisation and allocation timepoints

Mth-3-1/2: recruitment & enrolment					Mth1-4: Intervention run						Mth5-8: Follow up				
wk3-4 Jun	Jul	Aug	wk3-Sep	wk4-Sep	wk1-Oct	wk2-Oct	wk3-4 Oct	Nov	Dec	Jan	Feb	Mar	Apr	May	Jun
**Data collection timepoint**			**T0/Baseline measure & randomise**<br>(4 weeks across intervention starts)<br>Randomisation close by 14th of ix mth 1					**T1**<br>Mid-intervention<br>end of mth 2		**T2**<br>End of intervention<br>end of mth 4				**T3**<br>4 month FU<br>End of mth 8<br>**Qualitative interview**	

10.6 Methods

10.6.1 Laboratory procedures

Not applicable.

10.6.2 Radiology or any other procedure(s)

Not applicable.

11 Safety events

11.1 Definitions

Adverse Event (AE) - any untoward medical occurrence in a participant that results in need of medical and/or mental health support, whether it is considered to be related to the intervention or not. These include clinical signs and/or symptom, or condition and/or an observation of a near incident. Examples include: emotional distress as experienced by a participant to an extent that medical/mental health support is indicated (This does not include pre-existing conditions recorded as such at baseline).

Serious Adverse Event (SAE) - any Adverse Event or untoward medical occurrence in a trial participant that can be wholly or partly to the intervention which resulted in any of the following:

- Results in death; or
- Is life-threatening (places the participant, in the view of the TSC, at immediate risk of death).

Important medical events that may not be immediately life-threatening or result in death or hospitalisation but may jeopardise the participant or may require intervention to prevent one of the outcomes listed in the definition of SAE will also be considered serious.

Unintended Consequences (UC) - For this online RCT of an eHealth intervention, we have identified another type of safety events: unintended consequences. We categorise incidents what do not fall within the definitions of AE or SAE but involve incidents that interrupt the participants' use of the interventions and/or cause any minor distress in the participants but that do not indicate needs of any medical support. Examples of UC include technical failure of the intervention platform, breach of ground rules by participants resulting in moderation or removal of certain contents on forums, either initiated by the online facilitator or due to concerns raised by other participants, or minor distress or query raised by participants (these include participants feeling emotionally aroused or identifying potentially or previously un-identified unmet needs or new query due to content of intervention).

Support mechanisms for safety events

The online intervention platforms are monitored during working hours by an online facilitator. All participants will have consented to participate in the intervention according to the ground rules which include not posting any inappropriate or potentially insensitive materials. In addition, there are two direct weblinks on the intervention platform home page where participants can get in touch with the study team for either: (1) general/emotional support; or (2) technical support.

Incidents of AE, SAE and/or UC will be identified through both active monitoring by the online facilitator and query/concerns raised by participants. All incidents will be recorded according to the procedures outlined in Section 11.2.

In terms of support mechanism actions, we have devised the following procedures:

- If an inappropriate post is spotted by the online facilitator, such post will be moderated or removed retrospectively, as early as possible. The online facilitator will get in touch with the participant who made the post to provide further guidance on the ground rules of participation.

- In the case of repeated breach of ground rules, a temporary ban or withdrawal of participants may be considered necessary.

- If the facilitator spots signs of emotional arousal or minor distress from any participants' posts even without any subjective concern raised, the facilitator will get in touch with the participants via email to establish their wellbeing and to check if any support is needed.

- In the case of participants getting in touch actively to seek technical support, they will receive an automatic message outlining Frequently Asked Questions (FAQ), including password or navigating problems, immediately on receipt of their query. In addition, our online facilitator will get in touch with the participant to offer support within three working days.

- In the case of participants getting in touch to seek general/emotional support, they will receive an automatic message that explains that our online facilitator will contact them within three working days to talk through their concerns and maybe to suggest/signpost further resources. The message also gives information on alternative support and/or helpline services, such as the NHS non-emergency number 111 and SANE helpline.

- In the events of information conveying serious concerns regarding the individual's wellbeing or safety, the study facilitator will consider reporting the concern to the appropriate authorities (such as GP, social services or mental health service depending on the services the individual is engaged with).

- These potential UC and AE are outlined in the Participant Information Sheet (PIS), as do the support mechanism offered by the study team.

11.2 Recording AE/SAE/UC

A record of all UC, AEs and SAEs, whether related or unrelated to the intervention will also be kept in the study team record, CRF and the Sponsor's AE Log JREOLOG0007.

All incidents will be recorded in detail including nature and description of incidents, and the process through which they are classified, and actions undertaken. This record will be presented to the core study team (JS, SG, CH and e-Learning Experts LW & AS) on an ad hoc basis as well as in the bimonthly core team meeting and to PRG which oversees the EFFIP project and meets three time a year.

In addition, these records will be presented to the TSC at each TSC meeting and on an ad hoc basis. The TSC Chair and the subject-expert member will review all SAEs within 14 days of the CI becoming aware of them, including actions undertaken.

11.3 Investigator responsibilities relating to safety reporting

Collection, recording and reporting of AEs and SAEs to the Sponsor will be done according to the Sponsor's Safety reporting for non-CTIMP studies SOP JREOSOP0033.

All SAEs will be recorded in the CRF, and the Sponsor's AE Recording Log JREOLOG0007. The AE Log will be sent to the Sponsor on request and every two months, and to the TSC prior to each TSC meeting or on an ad hoc basis, if indicated.

All SAEs will be reported both to the Sponsor via the JREO & REC using the SAE report form for research other than CTIMPs (non-CTIMPs) published on the HRA website. The CI will liaise with the local PI at any participating site to complete the SAE form which will be faxed both to the JREO on 02087250794 or E-mailed to adverseevents@sgul.ac.uk, within 48 hours of the Investigator becoming aware of the event, and via email to the relevant REC.

The Chief Investigator will respond to any SAE queries raised by the Sponsor as soon as possible. Follow up reports must continually be completed within acceptable time-frames and sent as detailed above until the reportable event is considered resolved. Events will be followed up until resolution; any appropriate follow-up information will be clearly marked as such and reported to the sponsor via the JREO as above in a timely manner. Full reports should be completed and submitted to REC within 15 days of the event.

11.4 Notification of deaths

Only deaths that are assessed to be caused by the trial intervention will be reported to the Sponsor. This report will be immediate.

12 Data management and quality assurance

12.1 Confidentiality

All data will be handled in accordance with the Data Protection Act 1998.

The participant's trial username ID (which will be made up of a pseudonym and numerical code) only, will be used for identification. The sponsor Subject ID log JREOLOG0002 can be used to cross reference participant's identifiable information.

All participants will consent to observe the ground rules in using the online resource, including respecting the confidentiality of all participants and the posts/discussion shared.

All data will be kept securely at all time. If and when IT equipment and mobile devises are involved, they will be encrypted with secure passwords and only accessible to the research team. Anonymised data will be stored in a secure location within the Sponsor's premises, separate from other information that carries data that may identify the participants, e.g. consent forms.

12.2 Data collection mechanisms

All data collection and analysis will be processed by the research team within St George's, University of London premises. Participant personal details that are collected for administrative purposes (e.g. consent form, payment record) will be stored securely. We use the Sponsor Subject ID log JREOLOG0002 to cross reference participant's identifiable information and their username/ID. Only the core research team will have access to this information.

The participants are enrolled onto the interventions (which are hosted in a secure server owned by the Sponsor) using their username/study ID. All evaluation data will be

collected as coded- and anonymised responses and free-text entries/qualitative feedback, directly recorded on our secure servers hosting the interventions-platform and the data collection online mechanism, based at the sponsor's site. Multiple security mechanisms such as WordFence are used to protect against possible unauthorised intrusion.

We anticipate to interview about 30 participants for their experiences in using COPe-support for the process evaluation study. Only the audio-recordings of the individual interviews bearing no personal identifiable data will be sent to an external transcribing agency for transcribing. This is an agency approved by St George's, University of London which is fully compliant with confidentiality and privacy codes. Written and anonymised transcripts (so that participant's or others' names if mentioned will be replaced by a codes) will be made from the recording and the analysis will be conducted on the transcribed materials. The recording will be destroyed after the transcripts are checked as accurate and will never be played in public.

All study data, will be anonymised and will be kept securely on encrypted computers and separately from the personal data. No names or other information that might identify the participants will be used in any publication or documentation arising from this study.

12.3 Incidental Findings

In the unlikely event of incidental findings raising concerns from participants, the strategies stated below for provision of support for the participants will be followed.

Carers who are participants of the RCT are not regarded as a vulnerable group as advised by carers' and mental health charities, such as SANE, The Carer's Trust, and our own patient and public involvement (PPI) consultation activities. Nonetheless, the CI (JS is a qualified mental health nurse) and members of the core research team who include mental health professionals, will offer support for any carer-participants should they experience any concerns related to the resource-content (some possible concerns include minor distress or unmet needs or potential unsatisfactory services identified). If signs of incidental findings are spotted in the forum posts made by the participants or queries raised by the participants themselves, the online facilitator will make contact with the participants within three working days. Through an online or phone discussion of the incidental findings, and should the carer-participant want additional support, the online facilitator will signpost them to various relevant agencies run by NGOs which accept self-referrals or are membership-based (such as Rethink Mental Illness, MIND, SANE, Carers UK, Carer's Trust), and/or suggest the participant to seek a referral from their GP for primary care support (e.g. through Talking Therapies, counselling).

Identification of incidental findings will be noted as well as the actions undertaken. We will handle and record the incidental findings according to our strategies in handling UC (Section 11.1) if they are classified as UC. Otherwise, incidental findings will be reported to the PRG meetings and discussed by the wider research team if there are values in reporting such incidental findings (anonymised) in our reports/publication.

12.4 Data handling and analysis

All outcome data are self-reports and input directly online by participants onto our web-based data collection platform (a Sponsor owned domain). These data will be exported by the CI from the platform as an MS excel file. Data will be checked to ensure that the output is accurate.

Usage data, such as participants' number of log-ins and page-views, and number of posts made on the forums, will be recorded by the eLearning experts (LW and AS) at the end of each intervention cohort, as an excel file.

After all the outcome measure data and usage data are collected, the CI (JS) will take responsibility in cleaning and checking the data, before transferring the data into SPSS or Stata for analysis. (see Section 14 for statistical analysis plan).

Further to the aforementioned individual interviews (Section 12.2), the CI will conduct the interviews via telephone or skype with the participants with the interviews recorded as MP3 or MP4 files. The recordings will be sent for transcribing into Word documents. The CI will check that the transcripts as MSWord documents are an accurate record of the recordings, before deleting the original recording. The CI, with a member of the core study team, will carry out the qualitative analysis using thematic framework analysis (39) with NVivo-10 (a software that helps organise qualitative data analysis). Thematic framework analysis involves three broad stages. First, initial themes are identified by "indexing" transcript. These themes then guided formation of a framework within which transcribed material is summarised. Key categories are then identified to help described the data. Finally, patterns of association are explored and explained. This method of analysis is chosen because it is suited to the analysis of large qualitative data-sets and enable feedback to the participants. Consultation with the wider research team including our Project Reference Group (including individuals with lived experience of psychosis and carers) will also be used to validate the analysis. If necessary, the wider research team will review the transcripts and review the analysis process.

We also plan to corroborate/triangulate the results from the qualitative process evaluation study with the usage data and the results from the outcome data analysis, for gaining further insights into any interactions between participants' subjective experience in using COPe-support and their objective outcomes and usage.

13 Archiving arrangements

The trial essential documents along with the trial database will be archived in accordance with the sponsor SOP JREOSOP0016. The agreed archiving period for this trial will be 10 years.

14 Statistical design

14.1 Statistical input in trial design

Dr Tao Chen and Dr Victoria Cornelius act as trial statisticians for this study.

14.2 Endpoints

14.2.1 Primary endpoints

The primary endpoint is defined as 20 weeks after randomisation and allocation at the end of intervention use. Participants will be invited to complete primary outcome and secondary outcome measures at the end of the intervention use (T2 or week 20 in Figure II and Figure III, Section 10).

14.2.2 Secondary endpoints

All other time points (10 weeks, 20 weeks and 40 weeks) are treated as secondary.

14.3 Sample size and recruitment

14.3.1 Sample size calculation

The sample size is calculated with reference to an earlier study testing an eHealth intervention on siblings (the E Sibling Project, 8) and the primary outcome measure data (i.e. WEMWBS). In order to detect a minimum difference in score of at least 3 in WEMWBS which is regarded as a meaningful change post-intervention (26) between treatment groups with 80% power and significance level 5%, based on an estimated SD of 9 from the E Sibling Project RCT baseline results, (21,22) 143 carer-participants per arm are needed. With estimated 20% attrition, additional participants (n = 74) leading to an overall sample size of n = 360 across both arms. Minimally Clinical Important Difference (MCID) will be taken to be a change of 3.(28)

14.3.2 Planned recruitment rate

With caseload data obtained from the lead NHS Trust (South West London & St George's Mental Health NHS Trust) and the Health and Social Care Information Centre (40), it is estimated that there are approximately 4,000 service users with a primary diagnosis of psychosis in each mental health trust. Based on recent publications about carers, (21,22) it is expected that approximately about 50% of service users have an eligible carer. With reference to the recruitment data from recent relevant research targeting family carers including the E Sibling Project, (20-22) we expect at least 25% of carers approached to consent to participate in the study, (i.e. about 500 per Trust).

On the basis of the above estimates and using all the collective recruitment optimisation knowledge from the local CRN network (as discussed in Section 8), collaborating Trusts and core study team, we plan to initially approach about 100 carers at each Trust, randomly selected from the eligible databases of carers (and/or caseload of service users). Further random selections can then be made periodically, if necessary, to ensure that both recruitment and retention targets will be met in the main trial.

The through-flow of potentially eligible carers along the recruitment process in the initial five - eight sites in the internal pilot is summarised in Table 1 below:

Table 1 – Through-flow of estimates for the internal pilot RCT

Recruitment sites	Caseload of SUs with psychosis	Meeting eligibility criteria (x50%)	Number of carers to be approached initially	Estimated number of recruited participants (x25%)
Trust 1	4,000	2,000	100	25
Trust 2	4,000	2,000	100	25
Trust 3	4,000	2,000	100	25
Trust 4	4,000	2,000	100	25
Trust 5	4,000	2,000	100	25
Trust 6	4,000	2,000	100	25
Trust 7	4,000	2,000	100	25
Trust 8	4,000	2,000	100	25
Potential pool of participants for the internal pilot RCT (150 required with 20% retention rate factored in)*				200

*recruitment estimates to be scaled up for the full RCT with 15 or more NHS Trusts.

We therefore plan to run the internal pilot over the initial 12 months with a recruitment target of 120 participants. The progression criteria from the internal pilot to the full trial at recruitment month 6 of the internal pilot RCT are:

- Eight study sites are set up; and
- At least 60 participants recruited (half of 120 required for the internal pilot RCT)

Further progression criteria to the full trial at recruitment month 12 of the internal pilot RCT are:

- An additional 60 participants recruited; and
- Retention of internal pilot RCT participants ≥80%.

Progression to the full RCT will be reviewed based upon the results of these criteria at the pre-set time points. Final planning of the full RCT will be reviewed to establish the indicated number of recruitment sites needed and expansion of advertisement and recruitment activities, in consultation with the Trial Steering Committee (TSC) and the collaborators.

14.4 Statistical analysis plan

14.4.1 Summary of baseline data and flow of patients

The principle of intention-to-treat (ITT) will be the main strategy of the analysis adopted for the primary outcome and all secondary outcomes. All randomised participants will be analysed in the arm they were randomised to regardless of subsequent adherence to the allocated intervention. A summary table will list the individual subjects sorted by treatment group and describe their protocol deviation/violation.

Baseline descriptive variables of participants will be summarised by treatment arm (but no significance testing between arms will be performed):

- Continuous variables will be summarised according to number of subjects with non-missing data (n), mean, standard deviation (SD), median, Q1 (lower quartile), Q3 (upper quartile), minimum, and maximum.

- Categorical variables will be summarised according to the absolute frequency and percentage of subjects (%) in each category level. The denominator for the percentages is the number of subjects in the treatment arm with data available, unless noted otherwise.

A flow chart will be drawn up showing the number allocated to each study arm and the number screened, enrolled, and followed-up in each study arm, and the number contributing to the primary analysis and per-protocol according to CONSORT 2010.

The number adhering to the intervention and use will be summarised. The number who withdraw or are lost to follow up will be summarised by treatment arm and time.

14.4.2 Primary endpoint analysis

The primary outcome will be analysed using a linear mixed model, with participant subject effects, and fixed effects for arm, time (10, 20, 40 weeks) and the randomisation stratification variable gender. In addition, in order to improve the precision of the estimate we will include variables: parent (Y/N); and living with the cared-for individual (Y/N). A time-by-arm

interaction in the model will be investigated. The model will be used to estimate the mean difference and 95% CI in WEMWBS between arms at the 20 week timepoint.

The unadjusted mean scores by time will be plotted with 95% CIs to display the results visually.

A sensitivity analysis of the primary outcome will also be performed to examine the use of 'compliers' using an instrumental variable approach with randomisation as the instrumental variable. A complier will be defined as participant who accessed the online intervention at least once in 10 or more separate weeks over the 20-week intervention time (i.e. number of log-ins). The intervention effect by different levels of 'use' (adherence) will also be explored.

14.4.3 Secondary endpoint analysis

For continuous outcomes a similar approach to modelling used for the primary outcome will be followed. For binary outcomes a generalised linear model with binary distribution and log link function will be used.

Transcribed qualitative data collected on participants' experience in using the intervention will be analysed using the framework analysis method, (42) assisted by NVivo (http://www.qsrinternational.com). This method of analysis is chosen because it is suited to the analysis of large qualitative data-sets and enable feedback to the participants. To ensure the analysis is grounded in the data, it will be performed in parallel to the qualitative data collection, so the developing themes and framework of analysis can be tested and validated in latter data.

14.4.4 Sensitivity and other planned analyses (if applicable)

The proportions of participants missing WEMWBS will be quantified and summarised by arm. Baseline characteristics of those missing outcomes will be compared to those with complete follow up by examining bivariate relationships and also with multivariable logistic regression. As the primary analysis uses a linear mixed model, and will include all participants with results valid under the assumption of Missing At Random (MAR), no multiple imputation will be undertaken for this trial.

'Use' will be measured through the number of time carers log into the system. Based on previous feasibility data we know that it is not reliable to use duration. The relationship between 'use' and intervention effect will be examined by estimating intervention effect in sub-groups using the primary model.

14.5 Interim analysis

Not applicable.

14.6 Other statistical considerations

A Statistical Analysis Plan (SNP) will be drawn up prior to final data extraction and signed off by the CI, Statisticians and TSC.

15 Committees involved in the trial

A Trial Steering Committee (TSC) provides overall supervision of the trial and ensures that it is being conducted in accordance with the principles of GCP and the relevant regulations. The Trial Steering Committee will agree the trial protocol and any protocol amendments and provide advice to the Investigators on all aspects of the trial.
Terms of reference of the TSC is attached in Appendix 1.

16 Direct access to source data

The Investigator(s)/institution(s) will permit trial-related monitoring, audits, REC review, and regulatory inspection(s), providing direct access to source data/documents. Trial participants are informed of this during the informed consent discussion.

17 Ethics and Research Governance requirements

The study team will apply for approval to conduct the RCT with the target participants largely recruited from NHS services, with Health Research Authority (HRA) and a NHS Research Ethics Committee (REC).

Before any site can enrol patients into the trial, the Principal Investigator must ensure written permission to proceed has been granted by that Trust Research & Development (R&D). The site must conduct the trial in compliance with the protocol as agreed by the Sponsor and, which was given favourable opinion by the REC and HRA approval.

It is the responsibility of the Principal Investigator (PI) at each site to ensure that all subsequent amendments gain the necessary approval.

Within 90 days after the end of the trial, the CI and Sponsor will ensure that the REC is notified that the trial has finished. If the trial is terminated prematurely, those reports will be made within 15 days after the end of the trial. The CI will supply an End of Study report of the clinical trial to the REC within one year after the end of the trial.

17.1 Annual Progress Reports (APRs)

The Chief Investigator will prepare the APR in accordance with JREOSOP0043. Following review by the Sponsor the report will be sent to the REC. The APR is due for submission annually within 30 days of the anniversary date on which the favourable opinion was given by the Ethics committee, until the trial is declared ended.

17.2 Notification of serious breaches of GCP and/or the protocol

Any Protocol Deviations, Violations will be documented using JREODOC0061, and entered onto the Sponsor's log JREOLOG0005. Potential Serious Breaches and Urgent Safety Measures will be recorded both on the Sponsor's Log JREOLOG0005 and processed according to JREOSOP0012 and where necessary JREOSOP0032.

A "serious breach" is a breach which is likely to effect to a significant degree:
(a) The safety or physical or mental integrity of the participants of the trial; or
(b) The scientific value of the trial.
The CI will notify the Sponsor immediately of any case where there exists a possible occurrence of a serious breach

18 Finance

This study, as part a bigger study entitled “EFFIP (E-support for Families and Friends of Individuals affected by Psychosis): A randomised controlled trial of a co-produced online intervention for carers, has secured full funding from National Institute for Health Research, under its Post Doctoral Research Fellowship (awarded to the CI, Dr Jacqueline Sin, reference: PDF-2015-08-035).

19 Insurance and indemnity

St George’s University of London holds insurance to cover participants for injury caused by their participation in the clinical trial. Participants may be able to claim compensation if they can prove that St George’s has been negligent. This includes negligence in the writing of the protocol, or selection of trial resources.

Participants may be able to claim compensation for injury caused by participation in this Trial without the need to prove negligence on the part of St George’s University of London or another party.

If a participant indicates that they wish to make a claim for compensation, this needs to be brought to the attention of St George’s University of London immediately. Failure to alert St George’s University of London without delay and to comply with requests for information by the sponsor or any designated Agents may lead to a lack of insurance cover for the incident.

20 IP and development policy

All Intellectual Property Rights and Know How (IP) related to the Protocol and the Trial are and shall remain the property of the Sponsor excluding pre-existing IP related to clinical procedures of any Hospital/University.

All contributors shall:

(1) assign their/its rights in relation to all Intellectual Property Rights and in all Know How, not excluded above to the Sponsor and at the request and expense of the Sponsor, shall execute all such documents and do all such other acts as the Sponsor may reasonably require in order to vest fully and effectively all such Intellectual Property Rights and Know How in the Sponsor or its nominee.

(2) shall promptly disclose to the Sponsor any Know How generated pursuant to this Protocol and not excluded above and undertake treat such Know How as confidential information jointly owned between it and the Sponsor.

Nothing in this section shall be construed so as to prevent or hinder and medical professional from using Know How gained during the performance of the Trial in the furtherance of its normal business activities, to the extent such use does not result in the disclosure or misuse of Confidential Information or the infringement of any Intellectual Property Right of the Sponsor.

21 Publication policy

Publication: “Any activity that discloses, outside of the circle of trial investigators, any final or interim data or results of the Trial, or any details of the Trial methodology that have not been

made public by the Sponsor including, for example, presentations at symposia, national or regional professional meetings, publications in journals, theses or dissertations.”

All scientific contributors to the Trial have a responsibility to ensure that results of scientific interest arising from Trial are appropriately published and disseminated. The Sponsor has a firm commitment to publish the results of the Trial in a transparent and unbiased manner without consideration for commercial objectives.

To maximise the impact and scientific validity of the Trial, data shall be consolidated over the duration of the trial, reviewed internally among all investigators and not be submitted for publication prematurely. Lead in any publications arising from the Trial shall lie with the Sponsor in the first instance.

21.1 Before the official completion of the Trial

All publications during this period are subject to permission by the Sponsor. If an investigator wishes to publish a sub-set of data without permission by the Sponsor during this period, the Steering Committee/the Funder shall have the final say.

Exempt from this requirement are student theses that can be submitted for confidential evaluation but are subject to embargo for a period not shorter than the anticipated remaining duration of the trial.

21.2 Up to 180 days after the official completion of the Trial

During this period the Chief Investigator shall liaise with all investigators and strive to consolidate data and results and submit a manuscript for peer-review with a view to publication in a reputable academic journal or similar outlet as the Main Publication.

- The Chief Investigator shall be senior and corresponding author of the Main Publication.
- Insofar as compatible with the policies of the publication outlet and good academic practice, the other Investigators shall be listed in alphabetic order.
- Providers of analytical or technical services shall be acknowledged, but will only be listed as co-authors if their services were provided in a non-routine manner as part of a scientific collaboration.
- Members of the Trial Steering Group shall only be acknowledged as co-authors if they contributed in other capacities as well.
- If there are disagreements about the substance, content, style, conclusions, or author list of the Main Publication, the Chief Investigator shall ask the Trial Steering Group to arbitrate.

21.3 Beyond 180 days after the official completion of the Trial

After the Main Publication or after 180 days from Trial end date any Investigator or group of investigators may prepare further publications. In order to ensure that the Sponsor will be able to make comments and suggestions where pertinent, material for public dissemination will be submitted to the Sponsor for review at least sixty (60) days prior to submission for publication, public dissemination, or review by a publication committee. Sponsor’s reasonable comments shall be reflected. All publications related to the Trial shall credit the Chief and Co-Investigators as co-authors where this would be in accordance with normal academic practice and shall acknowledge the Sponsor and the Funders.

22 Statement of Compliance

The trial will be conducted in compliance with the protocol, Sponsor's Standard Operating Procedures (SOPs), GCP and the applicable regulatory requirement(s).

The study conduct shall comply with all relevant laws of the UK country in which the study site is located including but not limited to, the Human Rights Act 1998, the Data Protection Act 1998, the Human Medicines Regulations 2012, ICH GCP, the World Medical Association Declaration of Helsinki entitled 'Ethical Principles for Medical Research Involving Human Subjects' (2008 Version), the NHS Research Governance Framework for Health and Social Care (Version 2, April 2005).

This study will be conducted in compliance with the protocol approved by the HRA & REC and according to GCP standards. No deviation from the protocol will be implemented without the prior review and approval of the Sponsor and REC (& HRA) except where it may be necessary to eliminate an immediate hazard to a research subject. In such case, the deviation will be reported to the Sponsor and REC as soon as possible.

23 List of Protocol appendices

Appendix 1 Terms of reference of Trial Steering Committee

24 References

1. NICE (2014) *Psychosis and schizophrenia in adults: Treatment and management* (CG 178). London: The British Psychological Society & The Royal College of Psychiatrists.
2. The Schizophrenia Commission (2012) *The Abandoned Illness: A Report from the Schizophrenia Commission.* London: Rethink Mental Illness.
3. Smith L., Onwumere J., Craig T., McManus S., Bebbington P. & Kuipers E. (2014) Mental and physical illness in caregivers: results from an English national survey sample. *British Journal of Psychiatry*. 205: 197-203.
4. Szmukler G.I., Burgess P., Herrman H., Benson A., Colusa S. & Bloch S. (1996) Caring for relatives with serious mental illness: the development of the experience of caregiving inventory. *Social Psychiatry & Psychiatric Epidemiology*. 31, 137-148.
5. Brown G.W., Monck E.M., Carstairs G.M. & Wing J.K. (1972) influence of family life on the course of schizophrenic disorders: a replication. *British Journal of Psychiatry*. 121, 241-258.
6. Ritchie J., Spencer L. & O'Conner W. (2003) Carrying out qualitative analysis. In: Ritchie J.& Lewis J. (eds) *Qualitative Research Practice: A Guide for Social Science Students and Researchers.* London: Sage. Pp. 219-262.
7. Pharoah F., Mari J., Rathbone J. & Wong W. (2010) Family intervention for schizophrenia. Cochrane *Database of Systematic Reviews Issue* 12. Art. No.:CD000088. DOI:10.1002/14651858. CD000088.pub3.
8. Xia J., Merinder L.B. & Belgamwar M.R. (2011) Psychoeducation for schizophrenia. *Cochrane Database of Systematic Reviews*. Issue 6. Art. No.: CD002831. DOI:10.1002/14651858.CD002831.
9. Care Act 2014 (available from http://www,legislation.gov.uk/ukpga/2014/23/contents/enacted) [Last accessed on 12th April 2017.]
10. Sin J., Gillard S., Spain D., Cornelius V., Chen T. & Henderson C. (2017) Effectiveness of psychoeducational interventions for family carers of people with psychosis: A systematic review and meta-analysis. *Clinical Psychology Review*. 56: 13-24.
11. Kuipers E., Onwumere J. & Bebbington P. (2010) Cognitive model of caregiving in psychosis. *British Journal of Psychiatry.* 196, 259-265.
12. Lobban F., Glentworth D., Haddock G., Wainwright I., Clancy A., Bentley R. et al. (2011) The views of relatives of young people with psychosis on how to design a Relatives Education and Coping Toolkit (REACT). *Journal of Mental Health*. 20, 567-579.
13. Sin J. (2013) Focus group study of siblings of individuals with psychosis: views on designing an online psychoeducational resource. *Journal of Psychosocial Nursing and Mental Health Services*. 51(6), 28-36.
14. Cucciare M.A. & Weingardt K.R. (eds) (2010) *Using Technology to Support Evidence-Based Behavioural Health Practices – A Clinician's Guide*. New York: Routledge.
15. Sin J., Jordan C., Barley E., Henderson C. & Norman I. (2015) Psychoeducation for siblings of people with severe mental illness. *Cochrane Database of Systematic Reviews* (protocol published in 2013, Issue 6. Art. No.: CD010540. DOI: 10.1002/14651858.CD010540)
16. Sin J. & Norman I. (2013) Psychoeducational interventions for families of individuals with schizophrenia: A mixed method systematic review. *Journal of Clinical Psychiatry*. 74(12), e1145-e1162.
17. Powell J., Chiu T. & Eysenbach G. (2008) A systematic review of networked technologies supporting carers of people with dementia. *Journal of Telemedicine and Telecare.* 14, 154-156.
18. Grover M., Naumann U., Mohammad-Dar L, Glennon D., Ringwood S., Eisler I. et al. (2011) A randomised controlled trial of an internet-based cognitive behavioural skills

package for carers of people with anorexia nervosa. *Psychological Medicine*. 41, 2581-2591.

19. Rotondi A.J. (2010) Schizophrenia. In Cucciare M.A. & Weingardt K.R. (Eds) *Using Technologies to Support Evidence-based Behavioural Health Practices – A Clinician's Guide.* New York: Routledge. Pp.69-90.
20. Lobban F., Glentworth D., Chapman L., Wainwright L., Postlethwaite A., Dunn G., Pinfold V., Larkin W. & Haddock G. (2013) Feasibility of a supported self-management intervention for relatives of people with recent-onset psychosis: REACT study. *British Journal of Psychiatry*. 203, 366-372.
21. Sin J., Murrells T., Spain D., Norman I. & Henderson C. (2016) Wellbeing, mental health knowledge and caregiving experiences of siblings of people with psychosis, compared to their peers and parents: an exploratory study. *Social Psychiatry & Psychiatric Epidemiology*. 51: 1247-1255.
22. Sin J., Henderson C. & Norman I. (2014) Usability of online psychoeducation for siblings of people with psychosis. *International Journal of Technology Assessment in Health Care*. 30(4), 1-7.
23. Sin J., Henderson C., Spain D., Cornelius V., Chen T. & Gillard S. (in press) eHealth interventions for family carers of people with long term illness: A promising approach? *Clinical Psychology Review.*
24. EFFIP project website (2017) Accessible via http://cope-support.org (last accessed on 26th July 2017).
25. Medical Research Council (2008) *Developing and Evaluating Complex Interventions: New Guidance.* Retrieved from: http://www.mrc.ac.uk/documents/pdf/complex-interventions- guidance/ (last accessed on 27th July 2017).
26. EFFIP Focus group study (under preparation)
27. COPe-support prototype usability evaluation study (under preparation)
28. Tennant R., Hiller L., Fishwick R., Platt S., Joseph S., Welch S., Parkinson J., Secker J. & Stewart-Brown S. (2007) The Warwick-Edinburgh Mental Wellbeing Scale (WEMWBS): development and UK validation. *Health and Quality of Life Outcome*. 5: 63.
29. Boutron I, Altman DG, Moher D, Schulz KF, Ravaud P; CONSORT NPT Group (2017) CONSORT Statement for Randomized Trials of Nonpharmacologic Treatments: A 2017 Update and a CONSORT Extension for Nonpharmacologic Trial Abstracts. *Annals Intern Medicine.* 167(1):40-47.
30. Sin J., Henderson C., Spain D., Cornelius V., Chen T. & Gillard S. (under review) eHhealth interventions for family carers of people with long term illness: A promising approach? *Clinical Psychology Review.*
31. Lazarus, P., & Folkman, S. (1984). *Stress, Appraisal and Coping*. New York: Springer Publishing Company.
32. Hoffmann T., Glasziou P., Boutron I., et al. (2014) Better reporting of interventions: template for intervention description and replication (TIDieR) checklist and guide. *BMJ.* 348:g1687.
33. Canvas (2017) Accessible via https://www.canvas.net (last accessed on 1st December 2017).
34. Evans-Lacko S., Little K., Meltzer H., Rose D., Rhydderch D., Henderson C. & Thornicroft G. (2010) Development and psychometric properties of the Mental Health Knowledge Schedule (MAKS). *Canadian Journal of Psychiatry*. 55: 440-448.
35. Szmukler G., Burges P., Herrman H., Benson A., Colusa S. & Bloch S. (1996) Caring for relatives with serious mental illness: the development of the experience of caregiving inventory. *Social Psychiatry & Psychiatric Epidemiology.* 31: 137-148.
36. Family questionnaire

37. Quirk A., Smith S., Hamilton S., Lamping D., Lelliott P., Stahl D., Pinfold V. & Andiappan M. (2012) Development of the carer well-being and support (CWS) questionnaire. *Mental Health Review Journal.* 17(3), 128-138.
38. Devlin N.J. & Krabbe P.F.M. (2013) The development of new research methods for the valuation of ED-5D-5L. *European Journal of Health Economics*. 14 (Suppl.1): S1-S3.
39. Ritchie J., Lewis J., McNaughton Nicholls C. & Ormston R. (Eds) (2014) *Qualitative Research Practice: A Guide for Social Science Students and Researchers (2nd Edn).* Los Angeles: Sage.
40. Health and Social Care Information Centre (2013) *NHS Outcomes Framework Indicators – December 2013 Release.* London: HSCIC.
41. Gupta S.K. (2011) Intention-to-treat concept: A review. *Perspectives in Clinical Research.* 2(3), 109-112.
42. Spencer, L., Ritchie, J., Ormston, R., O'Connor, W. & Barnard, M. (2014) Analysis: principles and processes. In: Ritchie, J., Lewis, J., McNaughton Nicholls, C. & Ormston, R. (Eds) *Qualitative Research Practice: A Guide for Social Science Students and Researchers.* London: SAGE Publications Ltd. Chapter 10.
43. White I.R., Horton N.J., Carpenter J., Pocock S.J. (2011) Strategy for intention to treat analysis in randomised trials with missing outcome data. *BMJ.* 342, d40.

Appendix 1 – TSC terms of reference

Trial Steering Committee
The EFFIP Project RCT
Terms of Reference

As required by the good practice guidelines for randomised controlled trials, the EFFIP project RCT (E-support for Families & Friends of Individuals affected by Psychosis: A randomised controlled trial of a co-produced online intervention), will be overseen by a Trial Steering Committee (TSC).

Role
The role of the TSC is to strengthen and assure governance of the EFFIP RCT by:

1. Supervising the overall programme on behalf of the funder (NIHR) and the programme Sponsor (SGUL) (noting that day to day management of the programme is the responsibility of the Chief Investigator (CI) and the core study team);
2. Monitoring progress in the delivery of the RCT against agreed milestones;
3. Providing expert advice to the CI and co-investigators that is independent of the core study team;
4. Advising on any proposed changes to the RCT in the light of unanticipated developments or the emergence of new evidence that impacts on trial plans;
5. Providing advice to the CI and the core study team, the funder, Sponsor, host institution and other parties on progress in the project as appropriate;
6. Providing a monitoring and advice function for the safety and wellbeing of the study participants. In view of the safety event data, the TSC will consider the need for establishing/appointing a separate DMEC if necessary;
7. Providing written reports to the funder justifying any requests for funding or time extensions made by the investigators, where deemed appropriate by the TSC;
8. Supporting the appropriate dissemination of the project's (especially the RCT) findings.

Function
The TSC will function as follows:

- The TSC will have a membership comprising an independent Chair and up to five independent members. One of whom will be a statistician, one a subject expert with a clinical background for the purpose of safety monitoring, and up to three bring a lived experience perspective on family/informal caregiving to the TSC;
- At each TSC meeting, up to three members of the core research team, including the CI, will attend. Additional members of the research team may attend meetings at the discretion of the Chair;
- Representatives of the project Sponsor will be invited to all meetings;
- The TSC will meet biannually, and more frequently if judged necessary, with a minimum of three of the independent members, including at least one with lived experience and one academic member, present at each meeting (from early 2018 to end of 2020 when the RCT will be set up and run);
- Responsibility for calling, arranging and minuting meetings rests with the CI, as agreed with the Chair;

- The TSC will report to Project Reference Group (who have overall monitoring and management function for the project) and the project Sponsor, following each meeting, in the form of reports or minutes as formally agreed by the TSC Chair, or by direct communication from the Chair in the event of serious concerns and disagreements.

To note:
Travel expenses and other justifiable costs related to membership of a TSC are reimbursable. No other payments or rewards will be given to professional members. Honoraria will be paid to lay members as agreed. All justifiable costs will be included within and met from the budget for the project.

EFFIP (E-support for Families and Friends of Individuals affected by Psychosis)

COPe-support (Carers fOr People with Psychosis e-support)

A randomised controlled trial of a co-produced online intervention for carers

Statistical Analysis Plan

Version 2.0

Date: 03/07/2020

Sponsor's Project Number: 18.0027
REC Reference Number: 18/SC/0104
IRAS ID: 240005
Protocol Version and Date: v2.0 dated 3rd July
Trial Registration for Current Controlled Trials unique registration number: ISRCTN 89563420

Authors: Victoria Cornelius PhD, Tao Chen PhD, Jack Elkes MSc,
Claire Henderson PhD, Steve Gillard PhD, Luke Woodham MSc,
and Jacqueline Sin PhD

Signatures

CHIEF INVESTIGATOR (CI):
Dr Jacqueline Sin
Chief Investigator
Phone: 02087255530 & 07817027035
Email: jasin@sgul.ac.uk

Signature:

Date: 14/07/20

Senior Statistician:

Dr Victoria Cornelius
Imperial Clinical Trials Unit, School of Public Health, Imperial College London,
Email: v.cornelius@imperial.ac.uk

Signature:
Date: 17/07/20

Trial Steering Committee Chair

Dr Bryn Lloyd-Evans
E-mail: b.lloyd-evans@ucl.ac.uk

Signature:
Date: 14/07/20

Document version history log

Date	Version	Changes made
10/10/2019	1.0	Version 1.0 created
03/07/2020	2.0	1) An additional missing data sensitivity analysis was added to explore the assumptions regarding MAR. This was added to be in line with good statistical practice. 2) Usage data is now not available on a daily level due to a change in the platform statistics- as a result and reference to 'daily usage' was updated to weekly

Contents

Glossary of Terms **5**

1. Description of the trial **6**

1.1 Trial design including blinding 6
1.2 Principal research objectives to be addressed 7
1.3 Method of allocation of groups 9
1.4 Duration of the Intervention period 9
1.5 Frequency and duration of follow-up 9
1.6 Visit windows 9
1.7 Data collection 9
1.8 Sample size estimation 10
1.9 Brief description of proposed analyses 10

2. Data analysis plan – data description **12**

2.1 Recruitment and participant flow 12
2.2 Withdrawals, loss to follow-up and missing data 12
2.3 Adherence (Usage) to allocated intervention arm 12
2.4 Additional non-allocated interventional support 13
2.5 Descriptive statistics for outcome measures 13
2.6 Adverse effect reporting 13

3. Data analysis plan – inferential analysis **15**

3.1 Analysis of primary outcome 15
3.1.1 Primary analysis 15
3.1.2 Sensitivity analysis to address the impact of missing data 16
3.1.3 Usage data 16
3.1.4 Mediation measures analysis 17
3.2 Analysis of secondary outcomes 18
3.3 Adverse effects 18
3.4 Subgroup analysis 19
3.5 Exploratory analysis 19
3.6 Statistical considerations 19
3.6.1 Missing baseline data 19
3.6.2 Missing outcome data 19
3.6.3 Missing items in questionnaires scores 19
3.6.4 Interim analysis and data monitoring 19
3.6.5 Multiple comparisons 20

4. Software **21**

5. Reference list **22**

Glossary of Terms

AE	Adverse Events
AEf	Adverse Effects
CACE	Complier Average Causal Effect
CfP	Cared-for person
COPe	**C**arers f**O**r People with **P**sychosis **e**-support
CONSORT	Consolidated Standards of Reporting Trials
CTU	Clinical Trials Unit
CWS	Carer Wellbeing & Support Questionnaire
ECI	Experience of Caregiving Inventory
EFFIP	E-support for Families and Friends of Individuals affected by Psychosis
EQ-5D-5L	EuroQol 5 Dimension 5 Level Survey
FQ	Family Questionnaire
IQR	Inter-Quartile Range
ITT	Intention-to-Treat
MCID	Minimal Clinically Important Difference
MAKS	Mental Health Knowledge Schedule
REML	Restricted maximum likelihood
SAE	Serious Adverse Event
SD	Standard Deviation
TSC	Trial Steering Committee
UC	Unintended Consequence
WEMWBS	Warwick–Edinburgh Mental Wellbeing Scale

1. Description of the trial

1.1 Trial design including blinding

The EFFIP trial is a randomised controlled trial (RCT) with two stages: an internal pilot RCT to test out the protocol and verify recruitment and retention; and a full RCT. The RCT uses a two-arm, individually randomised controlled superiority trial design comparing the online intervention (in addition to usual care) with a waitlist control (in addition to usual care). Participant pathway through the RCT is shown in the CONSORT diagram.

Figure 1: Trial flowchart

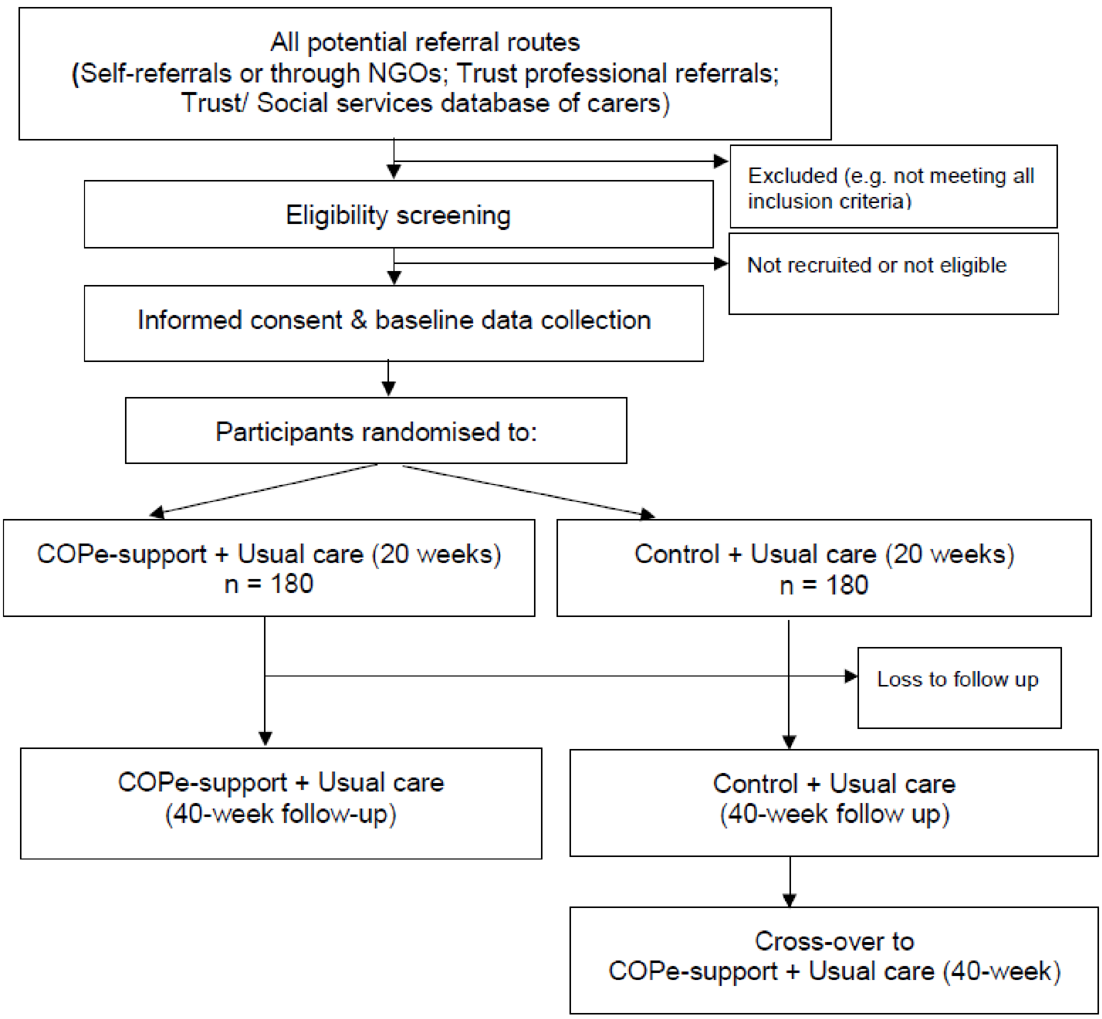

.

The study facilitator and participants are not blind to treatment intervention due to its nature. The study statistician(s) undertaking the primary analysis will be blind and will complete as much of the secondary analysis blind except for the adherence (usage) and mediation analysis where this is not possible.

1.2 Principal research objectives to be addressed

Primary objective:

The aim of the EFFIP project is to evaluate an internet-based multi-component support intervention for carers of individuals affected by psychosis, in promoting carers' mental wellbeing with a focus on helping them to gain essential knowledge and coping strategies to support the service users in their caring role.

Primary outcome measure:

Carers' mental wellbeing measured using the Warwick-Edinburgh Mental Wellbeing Scale (WEMWBS, 1) at 20 weeks (end of intervention).

Secondary outcome measures:

- Carers' mental health knowledge using Mental Health Knowledge Schedule (MAKS, 2)
- Carers' experience of caregiving using Experience of Caregiving Inventory (ECI, 3)
- Carer's quality of life using EQ-5D-5L (4)
- Carer's perceived support using Carer Wellbeing & Support Questionnaire (CWS, 5)
- Family relationship and communication as assessed by Family Questionnaire (FQ, 6)
- Carer's satisfaction with the intervention as a process evaluation outcome with post-use individual interview
- Online usage of the intervention and control
- Adverse effects to carers via safety monitoring i.e. adverse events recorded

Exploratory outcomes:

- Service use (e.g. statutory and voluntary services)

Table 1: Study Visits

Time point		STUDY PERIOD					
		Enrolment	Allocation	Post-allocation			Process evaluation
		T-1	T0	T1	T2	T3	T4
		Wk -18-0	Wk 0-2	Wk 10	Wk 20	Wk 40	Wk 41-45
**ENROLMENT**	Eligibility screening	X					
	Informed consent	X					
	Sociodemographic & caring data	X					
	Randomisation		X				
**INTERVENTIONS**	COPe-support			X	X	X	
	Waitlist			X	X	X	
**ASSESSMENTS**	Caring-related data (including health & social service use)		X	X	X	X	
	WEMWBS		X	X	X	X	
	MAKS		X	X	X	X	
	ECI		X	X	X	X	
	EQ-5D-5L		X	X	X	X	
	FQ		X	X	X	X	
	CWS		X	X	X	X	
	Perceived acceptability						X
	Usage data						X

T-1: Pre-randomisation; T0: baseline and randomisation; T1: mid-intervention; T2: end of intervention; T3: 40-week follow up; T4: after follow-up outcome data collection; WEMWBS: Warwick-Edinburgh Mental Wellbeing Scale; MAKS: Mental Health Knowledge Schedule; ECI: Experience of Caregiving Inventory; EQ-5D-5L: EuroQol 5 level version of EQ-5D; FQ: Family Questionnaire; CWS: Carer Wellbeing & Support Questionnaire.

1.3 Method of allocation of groups

Only consented participants who have completed baseline assessment will be entered into the randomisation process. Carers with completed baseline assessments will be individually randomised into the study to either COPe-support and usual care or to the control and usual care arm. Randomisation will be performed using permuted blocks of varied size, stratified by gender and cohort via an online bespoke system, developed and hosted by the King's CTU (http://www.ctu.co.uk).

1.4 Duration of the Intervention period

The duration of the study is 20 weeks (i.e. primary endpoint) but the total duration of the final follow-up period is 40 weeks.

1.5 Frequency and duration of follow-up

After screening and consent has been obtained baseline assessment (T0) will occur up to 2 weeks prior to intervention exposure. Participants will then be followed up for 40 weeks with visits for assessment at 10 weeks (T1), 20 weeks (T2) and the final follow up will be at 40 weeks (T3).

1.6 Visit windows

A visit window of 2 weeks will be targeted for participants, i.e. for T1 (10 weeks) the participants could be assessed between week 8 to week 12. Participants will be prompted for outcomes according to a planned visit schedule (see Table 1).

1.7 Data collection

All outcome data (apart from the process evaluation through individual interview) at all time points, are collected online through direct outcome measures inputs completed by participants through an online platform. The coding available for both the primary and secondary questionnaires/outcome measures will be used to translate answers into a numeric to calculate the total scores as appropriate.

Participants' views and experiences of using the intervention will be collected via individual interview through phone or internet. These will be conducted only after all the primary and secondary outcome data collection is completed.

Usage data of each participant will be extracted from the intervention (& control) platform after all the follow up data collection is completed.

All consented participants are assigned a unique ID and so do their outcome and usage data, hence to link the participants with their data.

1.8 Sample size estimation

The sample size is calculated with reference to an earlier study testing an eHealth intervention on siblings (the E Sibling Project, 7) and the primary outcome measure data (i.e. WEMWBS, 1). This study is powered to detect the minimum clinically important difference of 3 in WEMWBS which is considered a meaningful change post-intervention (1) between treatment groups. Using 80% power, significance level 5% and an estimated SD of 9 from the E Sibling Project RCT baseline results (8), 143 carer-participants per arm are needed. With estimated 20% attrition, additional participants (n = 74) leading to an overall sample size of n = 360 across both arms.

1.9 Brief description of proposed analyses

The data analysis will be performed by the trial statistician(s) who will be blind to randomised allocation for the primary and secondary outcome analysis. Further analysis of the usage data and exploration of mediation will not be blind as this is not feasible. Therefore, unblinded analysis will not occur until completion of all blind analysis has been performed and validated. The trial will be reported using the Consolidated Standards of Reporting Trials (CONSORT).

Baseline demographic characteristics and outcome measures will be summarised separately for each treatment arm and cohort. All continuous outcome measures will be summarised using means and standard deviations if approximately normally distributed or median (range, IQR) if the distribution is skewed. Distributions of continuous data will be explored using visual plots available, i.e. histograms and Q-Q plots, which will inform decisions about skewness, no formal testing will be performed. Categorical data will be described in terms of frequencies and proportions in each treatment arm and for each cohort.

Where missing data exists the pattern of this will be explored and its association with the primary and secondary outcomes. The proportion of missing will also be summarised for each treatment arm at each time point. Missingness in the baseline evaluations will be imputed to prevent loss of power (9). Imputation will use pooled data from all observations due to randomisation allowing baseline assessments and treatment allocation to be independent. Analysis of the primary outcome uses maximum likelihood estimation so is efficient for handling missing data under Missing-at-Random (MAR) assumptions. Sensitivity analysis for the primary outcome will explore the impact of departures from the MAR assumption. Although missing data will be described for secondary outcomes, no formal sensitivity analysis will be performed for secondary outcomes.

Analysis on efficacy outcomes will include adjustment for stratification variables, cohort (6 in total) and gender, as these have been shown necessary to include to maintain the correct type 1 error rate (10, 11). Additionally, for continuous outcomes, the outcomes measured at baseline will be included in regression analyses to increase power (12).

The primary outcome, WEMWBS, will be analysed using a baseline adjusted linear mixed model of the mean difference in score between treatment arms at 10, 20 and 40 weeks. The primary outcome is the mean difference in score at 20 weeks. The model will include baseline score, time point, treatment, a time point and treatment interaction, gender, parent (Y/N) and living with the cared-for individual (Y/N) as fixed effects with cohort and subject as random effects, even with only 6 cohorts there is enough for appropriate estimation (13). Model assumptions will be checked through post estimation plots of residuals and where the assumptions are not valid data transformations will be considered. Should the model not be valid alternative models will be used.

For all secondary outcomes, those which are continuous, will be analysed using a linear regression approach. The model will include treatment arm, baseline WEMWBS score, gender and cohort. The analysis will be performed separately at each of the 3 time points (10, 20 and 40 weeks), with the primary focus at 20 weeks. Adverse effects data will be captured in the reporting of AE data.

The total number and the unique number of participants experiencing an adverse effect (AEf) will be summarised for each treatment arm. Summaries of AEfs that were either Unintended consequence (UC) or Adverse Events (AE) (or both) will be tabulated in terms of number of events and number of participants. All outcomes of AEs will be described for each treatment arm and each cohort. If appropriate further analysis of AEfs will include performing a negative binomial or zero-inflated Poisson regression to estimate the relative risks between treatment arms. To further aid review graphs will be used to view AEfs over time through time-to-event plots. Regression analysis will be done for group adverse effects (AE and UC) and relative risk estimates will be calculated.

Usage data will be described separately for each arm. Summary statistics for continuous and categorical data as appropriate with either mean and SD or median and IQR for continuous variables. Usage data will be described in terms of “Users” and “Non-users” as well as in terms of “Compliers” and “Non-compliers” for the COPe arm only as defined in section 2.3. A sensitivity analysis of the primary outcome will be performed using a CACE analysis to compare mean WEMWBS score at 20 weeks, using the definition of “Compliers” for participants in the COPe arm.

For all analyses performed 2 sided 95% confidence intervals and their corresponding p-values will be presented and interpreted. For the primary outcome estimates and 95% confidence intervals will also be presented graphically separately for each treatment arm over the 4 timepoints.

2. Data analysis plan – data description

2.1 Recruitment and participant flow

The number of participants randomised will be summarised by intervention arm and cohort. Data by trust will also be described. To summarise the patient flow through the trial a Consolidated Standards of Reporting Trials (CONSORT) flow chart will be constructed (14). This will include the number of screened participants, number of eligible participants randomised into the trial, number of participants actively withdrawing from data collection, the number completing the 10, 20 and 40 week assessment, and the number included in the analyses.

2.2 Withdrawals, loss to follow-up and missing data

The number actively withdrawing from data collection in the trial will be reported by intervention arm and time point along with reasons for withdrawal. The proportions of participants with missing outcome values will be summarised in each arm and at each time point the measurement is planned.

2.3 Adherence (Usage) to allocated intervention arm

We will describe the ‘usage’ of the intervention for participants in the intervention arm. We will also describe how often the control participants accessed their ‘non-active’ website information using the same usage metrics where suitable.

The following definitions of usage are for ongoing monitoring only (TSC report):

Users: are participants who have logged in once and view more than 1-page (i.e. the home page by default following login activation) in either arm

Non-users: are participants who have not logged in or only logged in once and viewed 1-page

The following definitions of usage are for **study analysis:**

Complier is someone who has logged in twice or more AND are in the intervention arm. It will not be possible to determine directly the participants in the control who are ‘compliers’ as they do not have access to the active intervention, and therefore not observable.

Usage for both arms will be defined and examined in a number of different ways:

1. Number of logins on separate weeks per participant (both arms)
2. Average page views per weekly logins per participant (both arms)
3. Total time spent on the platform per participant (both arms)
4. Average time spent per page view on the platform per participants (both arms)
5. Total number of posts per participant to the peer-to-peer forum (intervention arm only)
6. Total number of posts per participant made to the Ask the Experts forum (intervention arm only)

We will look at ‘usage’ descriptively for both arms (where appropriate) but the main focus of the descriptive usage analysis will be for the intervention arm.

We will also undertake an analysis to adjust the estimate taking account of 'usage'. This will be undertaken using a complier adjusted causal effect (CACE) analysis. This approach can only include usage information in the intervention arm as the equivalent usage in the control participants is not observable (as the control intervention contains no active ingredients).

2.4 Additional non-allocated interventional support

As information on use of external active support outside of the trial is collected as part of outcome data collection, we will calculate the proportion of participants undertaking/receiving this. Data have been collected as binary - either received additional support or not

Definitions of non-allocated interventional support:

**Statutory services**	▪ Seeing a GP (Y/N)<br>▪ Carer support worker from Trust or social service (Y/N)<br>▪ Having a carer assessment conducted by Trust or social services personnel (Y/N)<br>▪ Having counselling or psychotherapy including cognitive behaviour therapy or counselling provided by Talking Therapies or other providers (Y/N)<br>▪ Seeking support from a care coordinator from the mental health service which supports the cared-for individual (Y/N)<br>▪ Seeking support from a social worker or personnel from the local authority/social service (Y/N)
**Voluntary services**	▪ Carer support group run by charity (Y/N)<br>▪ Peer-led support network (Y/N)<br>▪ One to one support provided by voluntary sector. (Y/N)
**Online or printed bibliotherapy**	▪ MOOC learning on psychosis or mental health-related subjects (Y/N)<br>▪ Information booklet/self-help books (Y/N)<br>▪ Online resource other than COPe-support, e.g. a charity website (Y/N)
**Other**	▪ Other support services for carers not specified above (Y/N)

As well as summarising each variable by arm we will also examine these four components aggregated. These will be summarised by the number of items per participant.

2.5 Descriptive statistics for outcome measures

Descriptive statistics will be presented for all outcome measures at 0, 10, 20, 40 weeks and usage data by intervention arm and for each cohort. Only participants with recorded outcomes will be used to calculate the summary measures.

2.6 Adverse effect reporting

Adverse effects (AEf) - any untoward medical occurrence in a participant that results in need of medical and/or mental health support, whether it is considered to be related to the intervention or not. These include clinical signs and/or symptom, or condition and/or an observation of a near incident. Examples include: emotional distress as experienced by a

participant to an extent that medical/mental health support is indicated (This does not include pre-existing conditions recorded as such at baseline).

Unintended consequence (UC): that cover incidents that interrupt the participants' use of the intervention (or control) and/or cause minor distress below the threshold of AE (e.g. forgetting their logins, realising unmet needs).

Serious Adverse Event (SAE) - any Adverse Event or untoward medical occurrence in a trial participant that can be wholly or partly to the intervention which resulted in any of the following:

- Results in death
- Is life-threatening (places the participant, in the view of the TSC, at immediate risk of death)
- Requires or prolongs hospitalisation

Coding UC, AE, and SAE: Events will be coded using terms of the clinical investigator choosing as outlined in the protocol, no dictionary will be used. All records of events will be reported to the study core team and the TSC for consensus on coding.

Adverse effects, which are unintended consequences and serious/non-serious adverse events will be summarised by type (UC, adverse events (AE) or serious adverse events (SAE)), and by treatment arm. UC and AE will be tabulated by intervention arm for both the number of events and the number of participants with the type of event.

No hypothesis testing will be undertaken for adverse event outcomes.

3. Data analysis plan – inferential analysis

3.1 Analysis of primary outcome

Outcome Definition: Carers' mental wellbeing measured using the Warwick-Edinburgh Mental Wellbeing Scale (WEMWBS) at 20 weeks (end of intervention).

3.1.1 Primary analysis

The ITT population for the trial will be all participants who were randomised and supplied at least one post-randomisation measure at 10, 20 or 40 weeks.

The primary outcome will be analysed using a linear mixed model. The model will include participant and cohort as random effects and will have fixed effects for arm, time (10, 20, 40 weeks) and the randomisation stratification variables gender as well as baseline score and a time-by-arm interaction. In addition, in order to improve the precision of the estimate we will adjust for variables parent (Y/N) and living with the cared-for individual (Y/N). The model's assumptions about random effects distributions and residuals will be investigated. If assumptions are poorly met then transformation will be considered.

The model will be used to estimate the mean difference and 95% CI in WEMWBS between arms at the 20-week time point (i.e. primary endpoint) and the main conclusion of the trial will be based on this time point. We will also report the intervention effect at week 10 and 40. The model will be fitted using REML.

With cohort and subject as random effects, where Y_{ijk} denotes the WEMWBS measurement for participant i at time j from cohort k, the primary analysis model will be:

$$Y_{ijk} = \beta_0 + \beta_1 Treat_i + \beta_2 \mathrm{WEMWBS}_{i0} + \beta_3 t_{20} + \beta_4 t_{40} + +\beta_5 t_{20} * Treat_i + \beta_6 t_{40} * Treat_i + \beta_7 PARENT \; + \beta_8 LIVINGIN_i + \beta_9 gender_i + a_k cohort_k + b_i participant_i + e_{ij}$$

For

j = 3 time points (week 10, 20, and 40), i = 360 participants,
Treat_i: dummy variable for intervention ($Treat = 0$ or 1) for participant i
$WEMWBS_{i0}$: baseline WEMWBS for participant i
$LIVINGIN_i$: living with the cared-for individual (Y=1 or N=0) for participant i
$cohort_k$: dummy cohort variable to be included (k=1,2 3,4,5,6) for participant i

t_x: dummy variable for time (= 0 or 1) at time point x weeks. E.g. week 10 is represented by $t_{10} = 1$ and $t_{20} = 0$ and $t_{40} = 0$
$a_k \sim N(0, \sigma_a^2)$, $b_i \sim N(0, \sigma_b^2)$, $e_{ij} \sim N(0, \sigma_e^2)$

Within the model a_k and b_i are random intercepts, at the cohort and participant level respectively. Each of a_k, b_i and e_{ijk} are assumed to follow normal distributions. An unstructured covariance matrix will be used. The treatment effect at 20 weeks, $\beta_1 + \beta_5$, will be of primary interest.

The addition of alternative relationships to cared-for individual will be explored as an additional analysis, using dummies for parent, spouse and sibling.

RELATIONSHIPi : other = 0, spouse is = 1, parent =2 for participant i included as a dummy variable.

The following STATA code will be used:

```
xtmixed y WEMWBS_Base LIVINGIN PARENT Gender i.treat##i.timepoint
        || subject:, nocons cov(unstr)
        || cohort:, nocons cov(unstr) reml

lincom (2.treat + 2.timepoint + 2.treat#2.timepoint) – (1.treat + 2.timepoint +
1.treat#2.timepoint)
```

The unadjusted mean scores by time will be plotted for each arm with 95% CIs to display the results visually.

3.1.2 Sensitivity analysis to address the impact of missing data

Every effort will be made to obtain follow up data for all participants including those that stop treatment. The number and proportion of participants missing WEMWBS by visit will be tabulated (see Section 1.9). The primary analysis methods outlined above employ maximum likelihood estimation and thus are efficient for handing missing outcome data under a Missing-at-Random (MAR) assumption. That is, the primary analyses will assume the probability of missing data is not dependent on the values of the unobserved data themselves, conditional on the observed values of the variables included in the analysis models.

Sensitivity analysis for WEMWBS we will explore the impact of departures from the main MAR assumption using a pattern-mixture MI approach (15). Imputation under MAR will initially be performed separately within each treatment arm using chained equations following the guidance suggested by White et al (16). Imputations will then be modified to reflect departures from the MAR assumption. Variables used in the imputation model will be those in the primary analysis model and additional auxiliary variables that were identified as strongly associated with WEMWBS from previous work. These are age of the cared-for person (CfP), ethnicity, relationship with CfP, marital status, duration of care and an interaction between age of CfP and relationship with CfP.

For WEMWBS we will investigate the impact of poorer response than that predicted by MAR for participants with missing data. To do so we will define δ as the postulated mean difference in the WEMWBS score between the observed and unobserved cases. For each participant in each intervention arm we will then modify the MAR imputed observations accordingly by δ. This δ is defined as a poorer WEMWBS score by the minimal clinically important difference (MCID) of 3 (17). Imputed data sets will be analysed using the primary analysis model outlined in Section 3.1.1. Results will be combined across imputed data sets using Rubin’s rules. For this sensitivity changes by δ will be considered for all participants with missing data, as well as informative missing in one arm only, and repeated for both arms. For each MI analysis 50 imputations will be run.

3.1.3 Usage data

We will summarise usage by arm and for each cohort separately and overall. While these summaries are of interest as outcomes in themselves to a certain extent the main focus will be on obtaining descriptive statistics for the intervention use and an estimate of the

intervention effect adjusted for usage. This will be achieved through a complier adjusted case analysis treating ‘usage’ as both binary and continuous variable.

A sensitivity analysis of the primary outcome will also be performed to examine the use of ‘compliers’ using a complier average causal effect (CACE) analysis using a two-stage least squares instrumental variable approach with randomisation as the instrumental variable. The definition as complier can be seen on page 12. The intervention effect by different levels of ‘use’ (adherence) will also be explored using the definitions of ‘usage’ on page 12. We will also categorise as ordinal variable and use as a continuous measure for which we will report the intervention effect for a one unit increase in ‘usage’, in terms of the six usage items as outlined in p.12 and their standard deviation:

1. Number of logins on separate weeks per participant (both arms)
2. Average page views per weekly logins per participant (both arms)
3. Total time spent on the platform per participant (both arms)
4. Average time spent per page view on the platform per participants (both arms)
5. Total number of posts per participant to the peer-to-peer forum (intervention arm only)
6. Total number of posts per participant made to the Ask the Experts forum (intervention arm only)

This will be undertaken using the command ivregress 2sls in Stata.

3.1.4 Mediation measures analysis

If there is an intervention effect, we will explore if this will work through the mechanisms underlying the intervention and as hypothesised by the adapted Stress-Appraisal-Coping model applied in family caregiving (3). The three mechanisms are shown in the diagram below and are; ‘appraisal’ (i.e. cognitive perception of caregiving situation as the stressor); ‘perceived support’ (via use of the Peer Forum and satisfaction with support perceived), and ‘mental health knowledge’ (via psychoeducation provided by the intervention).

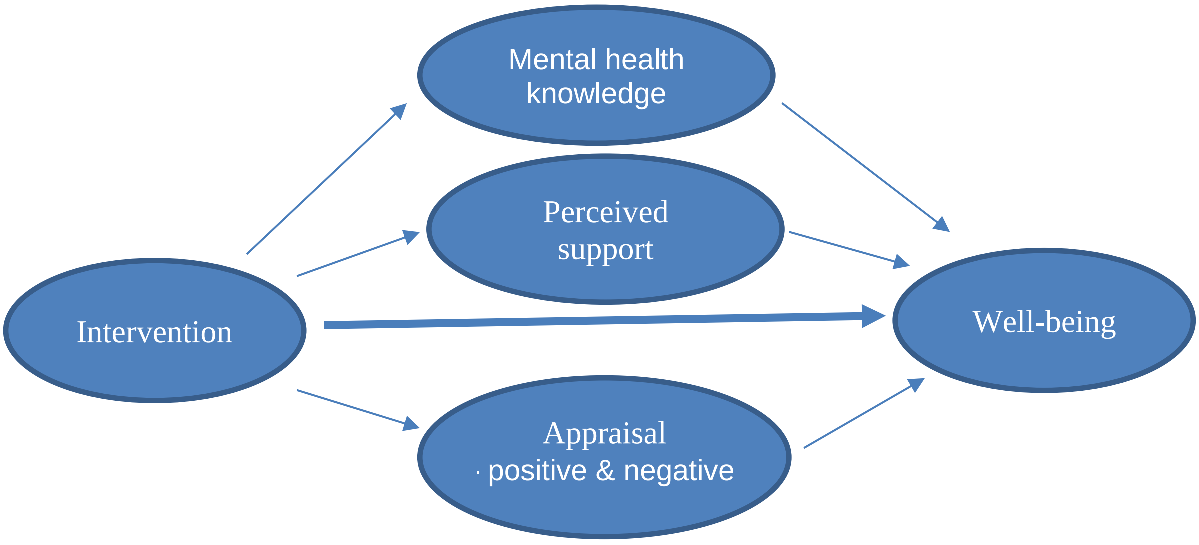

‘Appraisal’ will be measured using Experience of Caregiving Inventory (ECI), with two subscales for positive and negative (3). These will be examined separately.

‘Perceived support’ will be measured using the support subscale of Carer Wellbeing and Support Questionnaire (CWS, 5).
‘Mental health knowledge’ will be measured using the Mental Health Knowledge Schedule (MAKS, 2)

We will undertake mediation analysis using structural equation modelling approach. Wellbeing will be measured at 20 weeks and 40 weeks and mediators at all time points (i.e. 10, 20 and 40 weeks) but “controlled/adjusted” for previous level and/or baseline as indicative. We will also have values for mediators and outcome at baseline. The analysis will allow us to decompose the total observed intervention effect into mediated (indirect) and non-mediated (direct) components. We will fit each mediator in turn separately and then undertake a multiple mediation analysis. Initially we will use mediators at a lagged time point, i.e. mediator at 10 weeks and outcome at 20 weeks, lagged mediators will be explored to account for an anticipated time lag between intervention, mediation and outcome. We will then include all mediators and outcome at all time points (10, 20 and 40 weeks). While not displayed in the model depicted above we will include adjustment for baseline measurements of the mediators and the outcome well-being (18). Model goodness of fit will be assessed through comparing the saturated and baseline model and use of likelihood ratio tests, goodness of fit indices including AIC, BIC and Comparative Fit Index. The model will be re-specified if model assumptions are judged to not be met. This will be achieved through variable transformation or the use of alternative models.

3.2 Analysis of secondary outcomes

All secondary outcomes are listed below and are continuous. They will all be analysed at the primary 20-week time point and analysis will be repeated at 10 and 40 week time period as well. A linear regression model will be used. The model will include cohort, arm, and gender and baseline score.

- Carers’ mental health knowledge, assessed using Mental Health Knowledge Schedule (MAKS, 2)
- Carers’ experience of caregiving using Experience of Caregiving Inventory (ECI, 3)
- Carer’s quality of life using EQ-5D-5L score (EQ5D, 4)
- Family relationships and communication using Family Questionnaire (FQ, 6)
- Carer’s perceived social support using Carer Wellbeing & Support Questionnaire (CWS, 5)
- Carer’s satisfaction with the intervention as a process evaluation outcome with post-use individual interview (study devised interview topic guide)
- Online usage of the intervention and control (descriptive analysis only)

3.3 Adverse effects

Outcome definition: Adverse effects, defined as the collection of adverse events, unintended consequences, or serious adverse events at the level of the investigators choosing, at described in the study protocol v1.2.

Analysis: As it is anticipated that there will be few AEs and SAEs and likely more occurrences of UC, these will be evaluated individually, but they may also be included in tabulations and plots if thought to aid review.

Where useful a negative binomial or zero-inflated Poisson regression will be used to estimate relative risks, risk differences and incidence rate ratios of non-serious events (i.e. UC) If suitable the timing of adverse events (using hazard plots) by treatment arm will be examined.

3.4 Subgroup analysis

We will compare the intervention effect separately for each of the following subgroups:

- Relationship - Parent / Spouse / Child / other relationships
- Accommodation - Live with CfP / do not live with CfP
- Hours of caregiving - <10 hrs / 10-19 hrs / 20-34hrs / 35-49 hrs / ≥ 50 hrs caring per week
- Carer work status - Carers who work or study / carers who do not work or study (including full or part-time basis)

We will obtain estimates for the difference by including variables in the model with an interaction term with treatment. We will visualise the estimates of effect using a forest plot.

3.5 Exploratory analysis

Not applicable.

3.6 Statistical considerations

3.6.1 Missing baseline data

It is unlikely that missing baseline data will be problematic for the analysis as baseline outcomes is required to be collected prior to randomisation. However, if any missing baseline outcomes occur, to avoid a loss of power baseline values will be imputed with the mean outcome value calculated from the non-missing values using pooled data from both treatment groups. This technique improves the statistical efficiency in the estimation of treatment effect and is justifiable since randomisation ensures that baselines are independent of treatment group.

3.6.2 Missing outcome data

The primary analysis will use all observed outcome data and will be conducted under the MAR assumption and no multiple imputation will be used.

3.6.3 Missing items in questionnaires scores

The number (and %) with complete data will be reported for each outcome with multiple components. Where available we will follow the published questionnaire recommended guidance.

3.6.4 Interim analysis and data monitoring

No interim analyses will be performed. The Trial Steering Committee will review all adverse events reported during the trial.

3.6.5 Multiple comparisons

No multiplicity adjustments will be performed for the secondary analysis and results will be viewed as hypothesis generating.

4.0 Software

Stata will be used for data description and the main inferential analysis. Specific Stata commands which may be used the conduct the inferential analysis have been indicated in the relevant sections.

5 Reference list

1. Tennant R., Hiller L., Fishwick R., Platt S., Joseph S., Welch S., Parkinson J., Secker J. & Stewart-Brown S. (2007) The Warwick-Edinburgh Mental Wellbeing Scale (WEMWBS): development and UK validation. *Health and Quality of Life Outcome*. 5: 63.

2. Evans-Lacko S., Little K., Meltzer H., Rose D., Rhydderch D., Henderson C. & Thornicroft G. (2010) Development and psychometric properties of the Mental Health Knowledge Schedule (MAKS). *Canadian Journal of Psychiatry*. 55: 440-448.

3. Szmukler G., Burges P., Herrman H., Benson A.., Colusa S. & Bloch S. (1996) Caring for relatives with serious mental illness: the development of the experience of caregiving inventory. *Social Psychiatry & Psychiatric Epidemiology.* 31: 137-148.

4. Devlin N.J. & Krabbe P.F.M. (2013) The development of new research methods for the valuation of ED-5D-5L. *European Journal of Health Economics*. 14 (Suppl.1): S1-S3.

5. Quirk A., Smith S., Hamilton S., Lamping D., Lelliott P., Stahl D., Pinfold V. & Andiappan M. (2012) Development of the carer well-being and support (CWS) questionnaire. *Mental Health Review Journal.* 17(3), 128-138.

6. Wiedermann G., Rayki O., Feinstein E. & Hahlweg K. (2002) The Family Questionnaire: Development and validation of a new self-report scale for assessing expressed emtoion. *Psychiatry Research.* 265-279.

7. Sin J., Henderson C. & Norman I. (2014) Usability of online psychoeducation for siblings of people with psychosis. *International Journal of Technology Assessment in Health Care*. 30(4), 1-7.

8. Sin J., Murrells T., Spain D., Norman I. & Henderson C. (2016) Wellbeing, mental health knowledge and caregiving experiences of siblings of people with psychosis, compared to their peers and parents: an exploratory study. *Social Psychiatry & Psychiatric Epidemiology*. 51: 1247-1255.

9. White, I.R. and Thompson, S.G. (2005), Adjusting for partially missing baseline measurements in randomized trials. Statist. Med., 24: 993-1007. doi:10.1002/sim.1981

10. Kahan B.C. & Morris T.P. (2012) Reporting and analysis of trials using stratified randomisation in leading medical journals: review and reanalysis. *British Medical Journal*. 345: e5840.

11. Kahan B.C. & Morris T.P. (2013) Analysis of multicentre trials with continuous outcomes: when and how should we account for centre effects? *Statistics in Medicine*. 2(7):1136-49.

12. Senn S.J. (1989) Covariate imbalance and random allocation in clinical trials. *Statistics in Medicine.* 8(4):467-75.

13. Clark T.S. & Linzer, D.A. (2015) Should I use fixed or random effects? *Political Science Research and Methods.* 3(2): 399–408.

14. Moher D., Schulz K.F. & Altman D.G. (2001) The CONSORT statement: revised recommendations for improving the quality of reports of parallel-group randomised trials. *The Lancet*. 357(9263):1191-4.

15. Cro, S, Morris, TP, Kenward, MG, Carpenter, JR. Sensitivity analysis for clinical trials with missing continuous outcome data using controlled multiple imputation: A practical guide. *Statistics in Medicine*. 2020; 1– 28.

16. White, I.R., Royston, P. and Wood, A.M. (2011), Multiple imputation using chained equations: Issues and guidance for practice. Statist. Med., 30: 377-399.

17. Maheswaran H, Weich S, Powell J, et al. Evaluating the responsiveness of the Warwick Edinburgh Mental Well-Being Scale (WEMWBS): Group and individual level analysis. *Health and Quality of Life Outcomes* 2012;10(1):156.

18. Landau S, Emsley R. & Dunn G. (2018) Beyond total treatment effects in randomized controlled trials: Baseline measurement of intermediate outcomes needed to reduce confounding in mediation investigations. *Clinical Trials.* 15(3): 247-256.

Supplement Table 1: Summary of usage by participants across arms for the first 20-weeks

Usage Summary	Statistical Summary			
	N	Mean (SD)	Median (IQR)	Min - Max
**COPe-support**				
Activated, n (%)	175 (85.8%)			
No of weeks with login	173	5.1 (4.25)	4.0 (2.00 - 7.00)	1 - 19
Total pageviews	174	274.8 (406.13)	145.0 (36.00 - 353.00)	0 - 3198
Average pageviews per login	173	46.4 (39.14)	35.0 (19.33 - 61.07)	0 - 228
Total activity (minutes)	174	183.6 (355.56)	69.7 (17.28 - 176.12)	2 - 2648
Average time spent per pageview	171	0.9 (1.31)	0.5 (0.30 - 0.86)	0 - 10
Post on forums	174	2.3 (5.02)	0.0 (0.00 - 3.00)	0 - 30
Peer to Peer posts	175	1.0 (2.47)	0.0 (0.00 - 0.00)	0 - 17
Ask the Experts posts	175	1.3 (3.13)	0.0 (0.00 - 1.00)	0 - 21
**Control**				
Activated, n (%)	180 (88.7%)			
No of weeks with login	179	3.1 (2.49)	2.0 (1.00 - 4.00)	1 - 15
Total pageviews	180	77.7 (84.26)	49.5 (15.00 - 125.00)	0 - 549
Average pageviews per login	179	24.1 (19.73)	20.0 (10.00 - 32.25)	1 - 109
Total activity (minutes)	180	55.5 (161.48)	20.4 (9.05 - 38.78)	1 - 1504
Average time spent per pageview	179	1.1 (2.77)	0.5 (0.26 - 0.97)	0 - 32
Post on forums	-	-	-	-

Supplement Table 2: Sensitivity assessment of MAR assumption at 20-weeks follow-up

Sensitivity analysis	Estimated Difference (95% CI)
MI to include all participants	0.24 (-1.43; 1.91)
Controlled MI: delta = 3 to shift all those missing data in either arm	0.44 (-1.16; 2.05)
Controlled MI: delta = 3 to shift all those missing data in COPe-support	0.09 (-1.50; 1.69)
Controlled MI: delta = 3 to shift all those missing data in Attention Control	0.75 (-0.84; 2.34)
